# Defining the Cellular Environment in the Organ of Corti following Extensive Hair Cell Loss: A Basis for Future Sensory Cell Replacement in the Cochlea

**DOI:** 10.1371/journal.pone.0030577

**Published:** 2012-01-27

**Authors:** Ruth R. Taylor, Daniel J. Jagger, Andrew Forge

**Affiliations:** Centre for Auditory Research, The Ear Institute, University College London, London, United Kingdom; Federal University of Rio de Janeiro, Brazil

## Abstract

**Background:**

Following the loss of hair cells from the mammalian cochlea, the sensory epithelium repairs to close the lesions but no new hair cells arise and hearing impairment ensues. For any cell replacement strategy to be successful, the cellular environment of the injured tissue has to be able to nurture new hair cells. This study defines characteristics of the auditory sensory epithelium after hair cell loss.

**Methodology/Principal Findings:**

Studies were conducted in C57BL/6 and CBA/Ca mice. Treatment with an aminoglycoside-diuretic combination produced loss of all outer hair cells within 48 hours in both strains. The subsequent progressive tissue re-organisation was examined using immunohistochemistry and electron microscopy. There was no evidence of significant de-differentiation of the specialised columnar supporting cells. Kir4.1 was down regulated but KCC4, GLAST, microtubule bundles, connexin expression patterns and pathways of intercellular communication were retained. The columnar supporting cells became covered with non-specialised cells migrating from the outermost region of the organ of Corti. Eventually non-specialised, flat cells replaced the columnar epithelium. Flat epithelium developed in distributed patches interrupting regions of columnar epithelium formed of differentiated supporting cells. Formation of the flat epithelium was initiated within a few weeks post-treatment in C57BL/6 mice but not for several months in CBA/Ca's, suggesting genetic background influences the rate of re-organisation.

**Conclusions/Significance:**

The lack of dedifferentiation amongst supporting cells and their replacement by cells from the outer side of the organ of Corti are factors that may need to be considered in any attempt to promote endogenous hair cell regeneration. The variability of the cellular environment along an individual cochlea arising from patch-like generation of flat epithelium, and the possible variability between individuals resulting from genetic influences on the rate at which remodelling occurs may pose challenges to devising the appropriate regenerative therapy for a deaf patient.

## Introduction

Death of the sensory “hair” cells from the organ of Corti – the auditory sensory epithelium of the cochlea – is the major cause of sensorineural hearing loss. When hair cells die the non-sensory supporting cells that surround each one close the lesion created. In non-mammalian vertebrates, lost hair cells are then replaced by new ones. These new hair cells arise from the supporting cells, which are generally unaffected by those agents which kill hair cells. Two means of generating new hair cells have been identified in non-mammalian vertebrates: direct phenotypic conversion (non-mitotic transdifferentiation) of supporting cells into hair cells [1], [2], [3], [4]; and initiation of cell division amongst the supporting cell population with daughter cells subsequently differentiating into hair and/or supporting cells to restore the sensory epithelium [3], [5], [6], [7]. In mammals, there is a limited capacity for hair cell regeneration in the vestibular system of the inner ear [8], [9], [10], [11] which has been suggested to occur exclusively by phenotypic conversion [12], but there is no regeneration of hair cells in the mammalian cochlea. Consequently the hearing impairment resulting from hair cell death is permanent.

Recently, there has been some progress towards developing procedures that might enable replacement of lost hair cells in the mammalian cochlea. Broadly, there are three approaches. One is to attempt to induce conversion of supporting cells into hair cells. This might be achieved either through transfection with a gene, *ATOH1*, which encodes a transcription factor that has been shown to be necessary to initiate the differentiation of precursor cells into hair cells [13], [14], [15]; or through pharmacological manipulation of the Notch-Delta lateral inhibition system that is involved in cell fate determination during development [16], [17], [18]. The second approach is to try to stimulate cell division amongst supporting cells and derive hair cells from the daughter cells in a manner similar to that which occurs spontaneously in the inner ears of non-mammalian vertebrates. The third regenerative strategy is to use stem cell technologies to obtain cells that will differentiate into hair cells and incorporate these into the epithelium that exists after the original hair cells have been lost [19], [20], [21].

These strategies present challenges for attempting to induce regeneration in a system that does not regenerate spontaneously because each of them will require a cellular environment that is conducive to the production, differentiation and survival of replacement hair cells. Phenotypic conversion likely requires that supporting cells are relatively unspecialised. Encouraging proliferation may require that supporting cells de-differentiate so they become sensitive to signals that stimulate cell division. Stem cells, in addition to needing to be incorporated into the epithelium, will require an environment that nurtures and supports the differentiation of hair cells. The longer term survival and maintenance of hair cells will also require that the supporting cells provide the appropriate homeostasis. Mutations in a number of different genes that encode proteins involved in the physiological functioning of supporting cells lead to hair cell loss [22], [23], [24], [25], [26]. Thus, understanding the characteristics of supporting cells that remain after hair cell loss is crucial to identifying feasible regenerative procedures and the likelihood for long term survival of replacement hair cells.

The supporting cells within the organ of Corti are structurally and functionally specialised. The columnar supporting cells that are in contact with the hair cells contain bundles of closely packed microtubules, a specialisation that appears in the latter stages of cochlear maturation [27], [28]. The presence of microtubule bundles provides one identifying marker for the differentiated cells. One role for supporting cells is to assist in the maintenance of a low K^+^ - ion concentration around the bodies of the hair cells. Their plasma membranes consequently contain proteins involved in uptake of K^+^ ions, in particular Kir4.1 [29] and KCC4 [22]. Expression both of Kir4.1(D. Jagger and J Kelly, unpublished) and of KCC4 [22] is up-regulated during the latter stages of organ of Corti maturation as supporting cells acquire their morphological characteristics. The supporting cells are also specialised for the uptake of glutamate, the neurotransmitter released upon hair cell stimulation, to maintain low glutamate levels at the afferent synapses. The membranes of these cells contain the glutamate-aspartate transporter (GLAST) [30], [31]. GLAST expression increases over the latter stages of organ of Corti development [32]. Supporting cells of the organ of Corti are also extensively coupled by gap junctions [33], [34], [35] that potentially provide an intracellular route for the transfer of K^+^ out of the sensory epithelium. In the mature cochlea these gap junctions contain connexin (Cx)26, Cx30 or both in the same gap junction plaque [33]. Cx30 appears relatively late in cochlear development, as supporting cells become specialised [34], and during embryonic life another gap junction protein, Cx43, is expressed early on but is then down-regulated and it is absent from the mature organ of Corti [33], [36]. Labelling for these proteins provides identifiers for the presence of the functionally specialised cells, and changes in the patterns of expression of any of these proteins amongst supporting cells may act as indicators of cellular re-organisation organisation and, since many of them appear only as supporting cells reach maturity, they may also be useful as markers of de-differentiation.

Agents that cause acquired hearing loss such as noise trauma, exposure to ototoxins, or ageing, as well as the effects of mutations in a number of different genes that result in deafness, all cause a progressive loss of hair cells from the organ of Corti that is followed by progressive changes in its cellular architecture. Ultimately this can lead to the replacement of the normal columnar sensory epithelium with an apparently unspecialised epithelium, almost squamous-like in appearance, that has been referred to as a “flat” or a “monolayer” epithelium [37], [38]. Sections of the temporal bones of severely to profoundly deaf people often, but not always, show such an epithelium to have been present in the cochleae [39]. Severe to profoundly deaf patients are those who might be candidates for cell replacement therapies. It is therefore important to define the characteristics of this epithelium and how it is derived. Remarkably little is known of how the re-organisation from a specialised columnar sensory epithelium to the seemingly much less specialised one occurs. In this paper, we report findings from a mouse model of acquired deafness [30] in which we have examined events that follow the initial hair cell death and lesion closure effected by the supporting cells.

In an earlier paper we described a mouse model of acquired deafness [40] in which we examined the extent and mechanisms of hair cell death. In the work reported here, we examine the events that occur following hair cell loss and determine whether the proteins expressed for specific supporting cell function are retained. Furthermore, since there is considerable variability between human patients exposed to similar conditions in the extent of hearing loss, suggesting genetic factors may influence damage and tissue repair processes in the cochlea, we also took advantage of our development of a mouse model of induced hair cell loss to explore whether genetic background plays any role in the responses of the organ of Corti following extensive hair cell loss, by examining two different mouse strains with differing susceptibilities to both noise-induced and age-related hearing loss [41]. The aim of the study is to gain a detailed understanding of the cellular and likely physiological environment that exists after hair cells have been lost in order to be able to select the most appropriate strategy for the replacement of hair cells in the organ of Corti and thereby restore auditory function.

## Materials and Methods

Studies were made with two different strains of mouse: CBA/Ca and C57BL/6. C57BL/6 are more susceptible to noise-induced hair cell injury than CBA/Ca and exhibit early onset age-related hearing impairment at about 6–9 months of age whereas in CBA/Ca mice, natural onset of hearing loss occurs late in life at ca. 2 years of age [41]. The animals (120 in total) were obtained from our own breeding colonies. All work involving animals was performed in accordance with regulated, licensed procedures of the British Home Office (Project Licence number 70/6958) and was approved by the Animal Ethics Committee of UCL (Project Code 1276).

Hair cell loss was induced in animals 17–21 days old by a single treatment of 0.75–1 mg/g kanamycin (either Sigma or Gibco) dissolved in phosphate buffered saline (PBS) delivered sub-cutaneously, followed 45 minutes later by a single intraperitoneal injection of 0.05 mg/g bumetanide (Burinex Injection, Leo Laboratories) [40]. Cochleae were obtained for examination (after sacrifice according to Schedule 1 procedures of the British Home Office) at various times from 2 days to 6 months after treatment. Auditory bullae were isolated and the cochleae exposed. Fixative was gently perfused into the cochlea via an opening at the base, created by breaking the bone between the round and oval window, and one at the apex made by removing bone at the apical tip of the cochlea. The bullae were then immersed in fixative and fixation continued with slow rotation at room temperature. The cochleae of at least 3 different animals were prepared for each assessment undertaken at every time point.

### Immunohistochemistry

In preparation for immunohistochemistry, the cochleae were fixed in fresh 4% paraformaldehyde in PBS for 1–2 hours. They were then decalcified in 4% EDTA in PBS pH 7.3 for 48 h at 4°C. For whole mount preparations, the organ of Corti was dissected in segments from apex to base and the pieces transferred to PBS contained in one well of a 72 well dish for further processing. For frozen sections, the decalcified bullae with cochleae exposed were infiltrated with 30% sucrose in PBS overnight at 4°C then embedded in 1% low gelling temperature agarose (Sigma) in 18% sucrose with NaN_3_ (to prevent bacterial contamination). Agarose blocks containing the cochleae were oriented on support stubs so that sections parallel to the long axis could be cut and the blocks were frozen in liquid nitrogen. Sections 15 µm thick were obtained.

Whole mount samples and frozen sections were permeabilised by incubation in 0.5% Triton X-100 (Sigma) for 10 minutes at room temperature then exposed to a blocking solution consisting of 10% horse serum in PBS for 2 h at room temperature. After brief PBS washes the samples were incubated with the primary antibody in 100 mM lysine, 0.2% Triton X-100 in PBS overnight at 4°C. After several washes in PBS, the specimens were incubated in the fluorescently conjugated secondary antibodies for 2 h at room temperature. Phalloidin conjugated to either fluorescein isothiocyanate- (FITC), tetramethyl rhodamine isothiocyanatate (TRITC) or coumarin was added at 1 µg/ml to the secondary antibody solution. After several further washes the whole mounts were transferred to slides. Slides with the sections or the whole mounts were coverslipped using an antifade mountant containing 4′,6-diamidino-2-phenylindole (DAPI) to label nuclei (Vectashield; Vector Laboratories). Slides were first assessed by wide field fluorescence microscopy and selected samples examined and imaged using a Zeiss LSM 510 confocal microscope. Primary antibodies used are listed in Table 1.

**Table 1:** Primary antibodies used in this study.

Antigen	Host/type	Source	Dilution	Labelling
Cx26	Rabbit polyclonal (Gap 28H)	W H Evans, Cardiff University	1∶200	
Cx26	Mouse monoclonal	Zymed (Invitrogen, Paisley, UK)	1∶200	
Cx30	Rabbit polyclonal	Zymed (Invitrogen, Paisley, UK)	1∶200	
Cx43	Rabbit polyclonal	Sigma (Poole, UK)	1∶200	
Kir4.1	Rabbit polyclonal	Alomone Labs (Jerusalem, Israel)	1∶100	
KCC4	Rabbit polyclonal	Thomas Jentsch, Berlin University	1∶200	
KCC4	Goat polyclonal	Abcam (Cambridge, UK)	1∶200	
Acetylated tubulin	Mouse monoclonal	Sigma (Poole, UK)	1∶300	
GLAST	Rabbit polyclonal	Chemicon (Millipore UK, Watford)	1∶100	
Calretinin	Rabbit polyclonal	Thermo Scientific (Fisher, Loughborough, UK)	1∶100	Inner hair cells, neurons
Parvalbumin	Mouse monoclonal	Sigma (Poole, UK)	1∶100	Inner and outer hair cells
Prestin	Rabbit polyclonal	JF Ashmore, UCL	1∶200	Outer hair cells
Myosin7A	Mouse monoclonal (Developed by DJ Orten)	Developmental Studies Hybridoma Bank (maintained by Dept Biology, University of Iowa, under auspices of the NICHD)	1∶200	Inner and outer hair cells
F4-80	Rat monoclonal	Abcam (Cambridge, UK)	1∶200	Mouse macrophages
CD45 (leucocyte common antigen)	Rat monoclonal	Chemicon (Millipore, Watford)	1∶200	macrophages
CD91	Mouse monoclonal	Zymed (Invitrogen, Paisley UK)	1∶200	macrophages

### Electron microscopy

For electron microscopy the cochleae were fixed in 2.5% glutaraldehyde in 0.1 M cacodylate buffer with 3 mM CaCl_2_ for 2 h at room temperature or for 18 h at 4°C. They were then postfixed in 1% OsO_4_ for 2 hours at room temperature. Cochleae were decalcified, either after glutaraldehyde fixation or after post-fixation in OsO_4_, in 4% EDTA in 0.1 M cacodylate buffer pH 7.3 for 48 h. Decalcification at the latter stage produced more rigid specimens for dissection and from which the tectorial membrane was more easily peeled away from the organ of Corti in samples to be processed for scanning electron microscopy (SEM).

Cochleae to be examined by SEM were dissected to expose the organ of Corti. The segments were processed through the repeated thiocarbohydrazide-OsO_4_ procedure [42], dehydrated in an ethanol series and critical point dried from CO_2_. They were mounted on specimen support stubs using silver paint and then sputter coated with platinum. The specimens were examined in a JEOL 6700F cold field emission instrument, equipped with an in-lens detector, operating at 3 or 5 kV. Images were recorded digitally. To gain an appreciation of the three-dimensional organisation of the tissue, stereo-pairs of images of the same field tilted by 10° relative to each other were obtained and red/blue anaglyphs were prepared using AnalySIS software (Olympus).

Cochleae for thin sectioning prior to transmission electron microscopy (TEM) were processed intact without any dissection after decalcification and post-fixation. During dehydration in an alcohol series, at the 70% ethanol stage they were en bloc stained in a saturated solution of uranyl acetate in 70% ethanol overnight at 4°C before completion of dehydration and embedding the entire cochlea in plastic resin. Sections of the whole cochlear height, cut approximately parallel to the long axis, were obtained at several depths through the cochlea to enable assessment of the whole cochlear spiral [40]. Sections for light microscopy (1 µm thick) were taken at each location and stained with toluidine blue before a series of thin sections (80 nm thick) for TEM were cut. At least one of the thin sections at each location was mounted on a formvar coated single slot grid to enable uninterrupted examination of the section of the whole cochlear height. Thin sections were stained with uranyl acetate and lead citrate, and examined in a JEOL 1200EXII instrument operating at 80 kV. Digital images were collected with a Gatan CCD camera. Montages of the entire width of the organ of Corti were compiled from sets of overlapping imaged fields using the “Photomerge” function in Photoshop CS4 (Adobe Systems Inc, San Jose, CA, USA)

### Cochlear slice preparations

Slices of the viable cochlea were obtained as described elsewhere [34] from 21 day old animals that had been injected with kanamycin-bumetanide at 17 days of age, i.e. at 4 days after treatment. Studies of dye transfer between cells were performed as described previously [34] in slices maintained in and superfused with artificial perilymph (150 mM NaCl, 4 mM KCl, 2 mM MgCl_2_, 1.3 mM CaCl_2_, 8 mM NaH_2_PO_4_, 2 mM Na_2_HPO_4_, and 5 mMglucose, pH adjusted to 7.3 with NaOH). Individual supporting cells were patch clamped and Lucifer Yellow (LY) and neurobiotin (NBN) injected into them via the patch clamp electrode during whole cell patch clamp recording and the slice incubated at room temperature for 10 minutes. The slice was then fixed in 4% parafomaldehyde. To detect NBN, slices were permeabilized (0.1% Triton X-100 for 40 min), blocked (0.1 M L-lysine, at 35°C for 40 min), and incubated for 2 h in Alexa Fluor 555-conjugated streptavidin (1∶200; Invitrogen, Carlsbad, CA). LY fluorescence is photobleached quickly during confocal microscopy, and its peak absorbance (428 nm) lies between the available laser lines. To overcome these limitations, LY was detected by incubating slices in anti-LY polyclonal antibody (Sigma) (1∶200 with 10% normal goat serum, overnight at 4°C). Slices were then incubated for 2 h in anti-rabbit IgG conjugated to FITC (DakoCytomation) (1∶200 for 2 h at 35°C).

### Image processing

All digital images were adjusted for optimal contrast and brightness and figures assembled using Adobe Photoshop software.

## Results

### The undamaged organ of Corti

**Figure 1:**
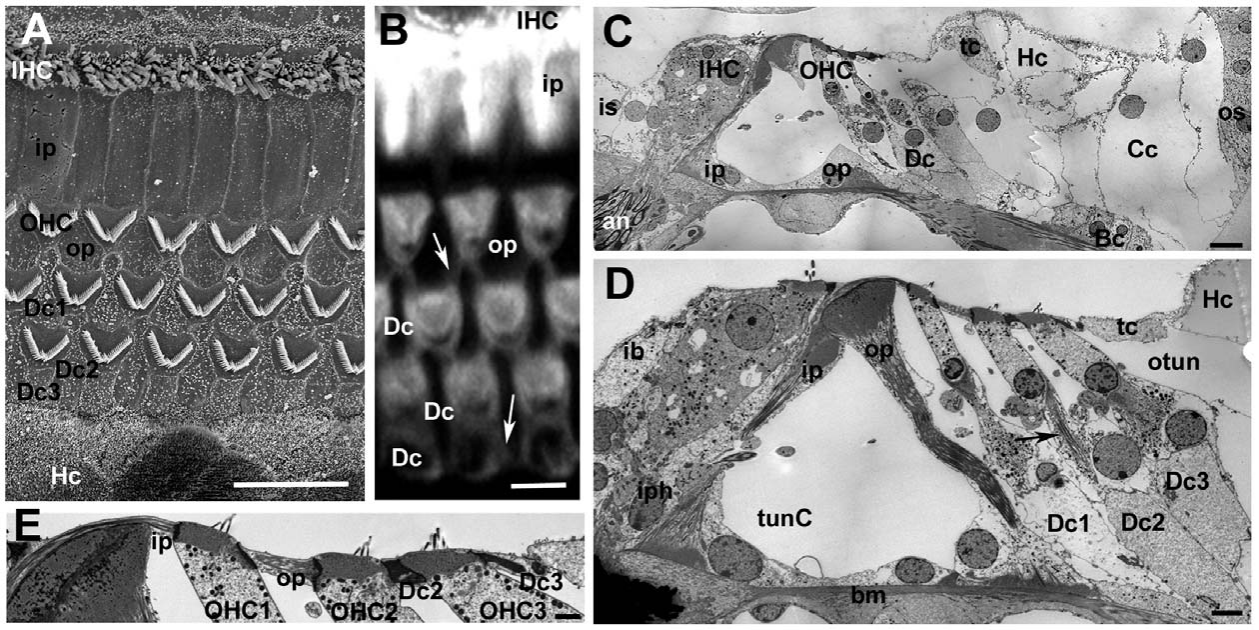
The organ of Corti of the mouse. Labelling for all figures: OHC = outer hair cell, IHC = inner hair cell; Dc = Deiters' cell, ip = inner pillar cell, op = outer pillar cell, Hc = Hensen's cell, Cc = Claudius cell, Bc = Boettcher's cell, os = outer sulcus, iph = inner phalangeal cell; ib = inner border cell, is = inner sulcus; tunC = tunnel of Corti, otun = outer tunnel; bm = basilar membrane. **A**. SEM of the apical surface of the organ of Corti (the “reticular lamina”). Each hair cell is separated from its neighbours by intervening supporting cells. The luminal surfaces of the Hensen's cells (HC) are distinguished by numerous microvilli. Scale bar: 10 µm. **B**. Confocal projection series image at the level of the reticular lamina of phalloidin-FITC labelled whole mount preparation. Phalloidin labels filamentous actin of the hair cell stereocilia, and the cuticular plate beneath the hair bundle in the apical cytoplasm of the hair cell, as well as the filamentous actin associated with the intercellular junctions. The arrow indicates the prominent, thick band of actin associated with the junctions between adjacent supporting cells in the OHC region. Scale bar: 5 µm. **C**. Montage of low power TEM images of thin section across the organ of Corti of the basal turn to show locations of all the cell types referred to in the text. The central strip of the sensory region containing the hair cells interspersed with specialised columnar supporting cells, is flanked either side with less specialised cells; the cuboidal inner sulcus cells to the inner side, and various cells types to the outer side. Scale bar: 10 µm. **D**. Thin section of sensory region of the organ of Corti showing some of the structural specialisations of the different supporting cell types. Arrow points to microtubule bundle in Deiters' cell. Bundles of microtubules are especially prominent in the pillar cells. Scale bar: 5 µm. **E**. The reticular lamina in thin section showing features at intercellular junctions. The head of the inner pillar and outer pillar cell are filled with microtubules, whereas the junction between an outer hair cell (OHC) and a Deiters' cell is characterised by microfilament assemblies, appearing as electron dense structures running the depth of the junction and widely into the supporting cell. Scale bar: 1 µm.

There are several excellent descriptions of the normal architecture of the organ of Corti (e.g. [43], [44]), but to provide a context for the changes that occur following loss of hair cells a description of the normal organisation is presented and illustrated in Figure 1.

The sensory strip within the organ of Corti consists of a single row of inner hair cells (IHC) and, usually, three (occasionally four) rows of outer hair cells (OHC), each hair cell separated from its neighbours by intervening supporting cells (Fig. 1A,B). The bodies of the supporting cells also intervene between the base of the hair cells and the basilar membrane, the extracellular matrix that underlies the entire organ of Corti (Fig. 1C,D). There are several types of less specialised cells to the outer (lateral) side extending across the basilar membrane to the outer sulcus cells at the lateral edge. On the inner side of the sensory strip are cuboidal cells that comprise the inner sulcus (Fig. 1C).

There are several different types of columnar supporting cells within the sensory strip. In the outer hair cell region there are three rows of Deiters' cells. Between the OHC region and that of the IHC are the outer and inner pillar cells (Fig. 1A,B, D). Surrounding the IHC are the inner phalangeal cells on the lateral (outer) side, abutting the inner pillar cell, and the inner border cell to the medial (inner) side adjacent to the inner sulcus. Deiters' cells and pillar cells comprise a cell body region, which sits on the basilar membrane and encloses the nucleus, and a thin phalangeal process that rises to the apical surface of the sensory epithelium where the head of the cell expands and is in junctional contact with hair cells and other supporting cells (Fig. 1A,B,D,E). Deiters' and pillar cells are characterised by the presence of organised bundles of microtubules that run from the base of the cell through the phalangeal processes (Fig. 1D). These are especially prominent in the pillar cells. The thinning of the cells that generates the phalangeal processes, which occurs during the latter stages of cochlear maturation concomitant with the formation of the organised microtubule bundles, creates large extracellular spaces within the organ of Corti: the spaces of Nuel between the bodies of the OHC; the tunnel of Corti created by the arch formed by the angled phalangeal processes of the outer and inner pillar cells; and the outer tunnel, between the third row of Deiters' cells and the tectal cell [44] on the outer side. Unlike the OHC, the bodies of IHC are in close contact with the bodies of the surrounding supporting cells.

Each Deiters' cell body encloses the basal end of the OHC and its innervation (Fig. 1D) and sends a phalangeal process angled along the longitudinal axis of the cochlear spiral in the apical direction to expand at the apical surface of the epithelium 1–2 OHC away from that whose body it encloses (Fig. S1A). The Deiters' cell head in each row lies behind (on the outer, lateral side of the apex of) the equivalent OHC (Fig. 1A,B). On the inner side of the OHC region, the head of the inner pillar cell extends across the top of the tunnel of Corti to contact the “front” (inner) side of the OHC in the first row (Fig. 1A,B,D,E), while the head of the outer pillar cell, which forms a buttress under the head of the inner pillar cell (Fig. 1D), extends outwards and contacts the front side of OHC in the second row as well as the heads of the first row of Deiters' cells (Fig. 1A,B,E). This organisation creates a regular mosaic at the apical surface of the organ of Corti, which is known as the reticular lamina.

The heads of the pillar cells, like their phalanges, enclose organised bundles of microtubules which extend laterally across the apical process of the cell to the junction with the OHC. Thus, the supporting cell side of the junction between 1^st^ row OHC with the inner pillar cell and that of the 2^nd^ row OHC with the outer pillar cell are identifiable by the presence of microtubules (Fig. 1E). Microtubule bundles are not prevalent in the heads of the Deiters' cells. At the level of the adherens junctions between adjacent Deiters' cells and between Deiters' cells and OHC there are prominent actin microfilament assemblies that label intensely with fluorescently-conjugated phalloidin (Fig. 1B). In thin sections, the actin assemblies associated with the adherens junctions of Deiters' cells appear as prominent, wide electron densities on the Deiters' cell side running the entire depth of the junctional contact the adjacent cell (Fig. 1E). These junctional features provide identifying markers for the cell types at the apical surface of the organ of Corti.

To the outside of the outermost row of Deiters' cells, is the tectal cell which covers the apical surface and forms the outer wall of the outer tunnel, and a Hensen's cell. The tectal cell does not reach down to the basilar membrane [44]; below the luminal surface the body of a Hensen's cell is in direct contact with the cell body of the third row Deiters' cell (Fig. 1D). On its outer side the tectal cell is in junctional contact at its luminal end with the Hensen's cell. The apical surface of the tectal cell is relatively smooth but Hensen's cells are characterised in surface views by densely packed short microvilli and in sections are seen to have a lighter cytoplasm and fewer organelles than Deiters' cells or tectal cells. Lateral to the Hensen's cells are the Claudius' cells, which extend to the outer sulcus at the outermost limit of the basilar membrane. Claudius' cells show even fewer cytoplasmic specialisations and organelles than do Hensen's cells, their cytoplasm appearing remarkably electron luscent in thin sections. In the basal coil of the cochlea, though less prominently at the cochlear apex, to the lateral side of the Hensen's cells, sitting on the basilar membrane and overlain by Claudius' cells are a group of Boettcher's cells (Fig. 1D). These cells are not exposed at the luminal surface because of the covering Claudius' cells. They are characterised by a dense cytoplasm, in stark contrast to the much lighter cytoplasm of the surrounding cells, and their apico-lateral membranes are extensively infolded and interdigitated.

### Loss of hair cells

The kanamycin-bumetanide treatment produces almost complete loss of all OHC within 48 hours in CBA/Ca mice [40]. This was also found with the C57BL/6 mice. The loss is initiated in the basal coils of the cochlea and progresses to the apical coil [40], [45]. Loss of IHC was delayed relative to OHC loss in both mouse strains. As noted previously [40], [45] a proportion of treated animals showed no hair cell loss; although not quantified these animals were almost always females, suggesting male animals were more susceptible to the treatment protocol than females.

### Initial repair of the organ of Corti by supporting cells

**Figure 2:**
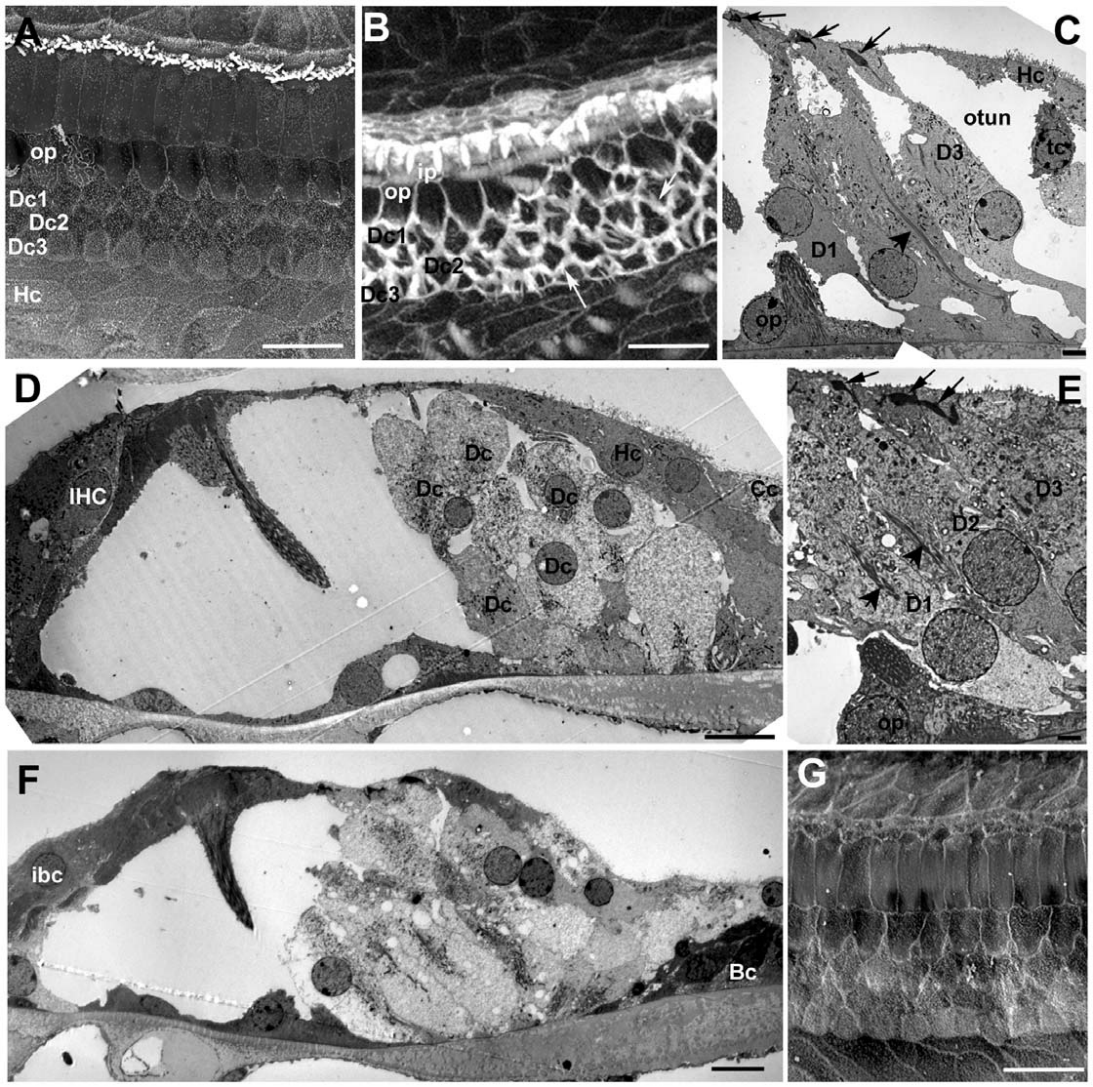
Repaired organ of Corti following hair cell loss. **A. CBA/Ca mouse; 7 days post-treatment.** All OHC lost but all IHC remain. The heads of Deiters' cells and of the outer pillar cells have expanded into the regions where OHC used to be. A regular pattern of cells is created at the epithelial surface. Scale bar: 10 µm. **B. C57BL/6; 14 days post-treatment.** Phalloidin-FITC labelled whole mount. Wide bands of actin persist at junctions between adjacent supporting cells. Inner hair cells have been lost and the heads of inner border and inner phalangeal cells have expanded to close the lesions. Scale bar: 10 µm. **C. CBA/Ca; 24 h post-treatment.** OHC lost and Deiters' cell heads have expanded to close the lesions at the luminal surface. There is some widening of the phalangeal processes but extracellular spaces are still open. Arrows indicate dense, microfilament assemblies at the junctions between the adjacent cells that are present in each of the three Deiters' cells. Larger arrowhead denotes microtubule bundle in Deiters' cell. Scale bar: 5 µm. **D. CBA/Ca; 14 days post-treatment.** Deiters' cells have generally expanded. The outer tunnel is closed but some extracellular spaces in the Deiters' cell region and the tunnel of Corti are still open. The overall architecture is preserved in the repaired epithelium. Scale bar: 10 µm. **E. CBA/Ca; 48 h post-treatment.** Expansion of the 3^rd^ row Deiters' cell closes the outer tunnel. Microtubule bundles (large arrowhead) and microfilament assemblies at the intercellular junctions are maintained in Deiters' cells. Scale bar: 5 µm. **F. CBA/Ca; 3 months post-treatment.** IHC as well as OHC are lost, but the tunnel of Corti is open and the cellular arrangement of the repaired organ of Corti is preserved. Scale bar 10 µm. **G. C57BL/6; 4 weeks post-treatment.** Both IHC and OHC are missing. The regular pattern of the cells at the surface of the repaired epithelium is maintained. Scale bar: 10 µm.

As described previously [46], [47], following the initial loss of OHC, the heads of the Deiters' cells and of the outer pillar cells expand and come into close contact creating so-called “scar” formations at the apical surface of the reticular lamina in the sites of the lost hair cell (not shown). The initial scar formations rapidly resolved to create a regular pattern of cells at the luminal surface of the repaired epithelium (Fig. 2A). Phalloidin labelling revealed distinctive wide bands of actin at the intercellular junction round the periphery at the apex of each Deiters' cell (Fig. 2B) and prominent microfilament assemblies at the adherens junctions in thin sections (Fig. 2C). Junctional actin bands were less prominent in outer and inner pillar cells. At the junction between the outermost Deiters' cell and the adjacent tectal cell the band of actin labelling on the Deiters' cell side was wider than on the tectal cell side, such that a relatively thinner actin band was present at this level. IHC often persisted in this repaired epithelium. With expansion at the apical end of the supporting cells the phalangeal processes of the Deiters' cell also expanded. Immediately following hair cell loss the spaces of Nuel and the outer tunnel were still open (Fig. 2C). Subsequently the outer tunnel and the spaces between Deiters' cells were filled by expansion of the Deiters' cell phalangeal processes (Fig. 2D, E), but the tunnel of Corti between the inner and outer pillar cells remained open, even where IHC were lost (Fig. 2F). Other than the expansion there was no indication of any other change in the morphological characteristics of Deiters' cells; their organised microtubule bundles persisted (Fig. 2E). The expansion of the supporting cell heads was accompanied by some change in cell architecture, however; as the head of the Dieters' cells expanded with loss of the OHC their phalangeal processes straightened, rising straight up from the cell body (Figure S1B,C) region rather than at an angle (Fig. S1A). In addition, in some Deiters' cells, the nucleus was relocated to a more apical position and the cell appeared to round up, seemingly losing contact with the underlying basilar membrane (Fig. S1D).

### Potassium transporters

**Figure 3:**
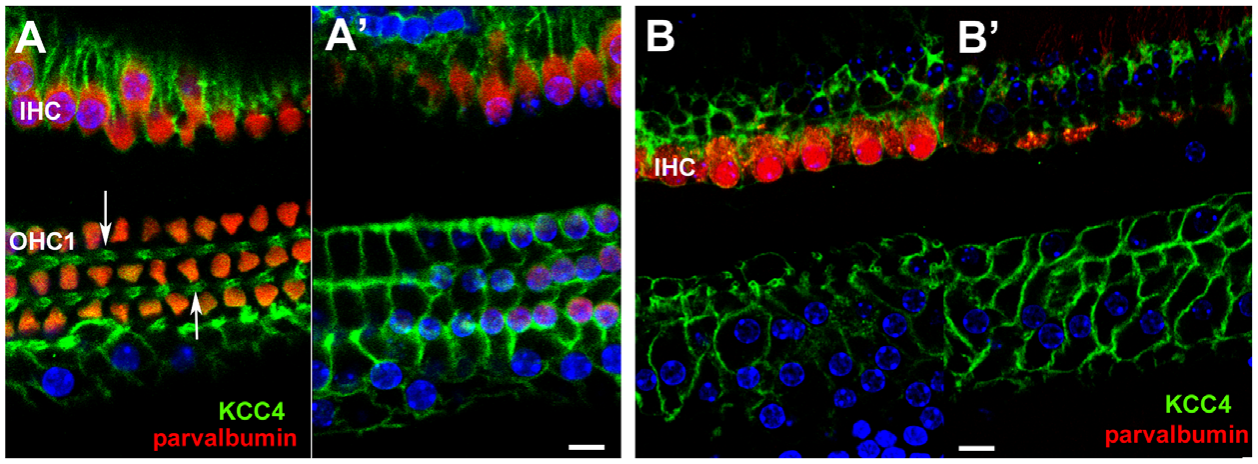
Immunolabelling for KCC4. **A,A'. Normal (undamaged) mature organ of Corti.** Confocal images of a whole mount preparation. A, at the level of the cell bodies of the OHC; A', at the level of the cell bodies of the Deiters' cells. Both IHC and OHC are labelled with antibodies to parvalbumin (red). KCC4 labelling (green) delineates the plasma membranes of Deiters' cells, both along the phalangeal processes (arrows in A) and around the cell bodies (A'). KCC4 is also present in the plasma membranes of the supporting cells around the IHC. There is no KCC4 labelling in the region of the pillar cells between the OHC/Deiters' cell region and that of the IHCs. **B,B'. 14 weeks post-treatment (CBA/Ca mouse). KCC4 is retained in Deiters' cells in the repaired epithelium.** Imaging levels for B and B' approximately the same as for A and A' respectively. Parvalbumin labelling (red) reveals complete absence of OHC but most IHC remain. KCC4 (green) is retained in the plasma membranes of Deiters' cells. The cells' shapes are delineated by the labelling and the variability is apparent. At the level of the phalangeal processes (B) some Deiters' cell are clearly expanded whilst others appear to have retained a thinned phalange. The bodies of many cells are also enlarged and irregular in shape (B') in comparison with the regularity seen in the normal organ of Corti (A'). There is no KCC4 labelling in the pillar cell region. Scale bars: 10 µm.

In the undamaged tissue, antibodies to KCC4 labelled almost the entire lateral plasma membranes of Deiters' cell, around the cell body and along the phalangeal processes between OHC (Fig. 3A A'). Such labelling thereby outlined the Deiters' cells. On the IHC side of the tunnel of Corti, KCC4 labelling was present along the lateral membranes of the inner border cells. Pillar cell membranes were not labelled by the KCC4 antibody, such that there was a distinct unlabelled area between the OHC and IHC regions across the area formed by the pillar cells and the tunnel of Corti. Following hair cell loss, KCC4 labelling was retained at a comparable intensity to that in the normal tissue and with no change in distribution (Fig. 3B B'). The entire depth of the lateral plasma membranes of Deiters' cells and of inner border cells was labelled. The consequent outlining of each Deiters' cell clearly revealed the expansion of the cells at the level of the phalangeal processes which brought neighbouring Deiters' cells closely adjacent in these regions, but also showed the variability in the shapes of the cells; some Deiters' cells retained a phalangeal-like process whereas others were greatly widened (Fig. 3B).

**Figure 4:**
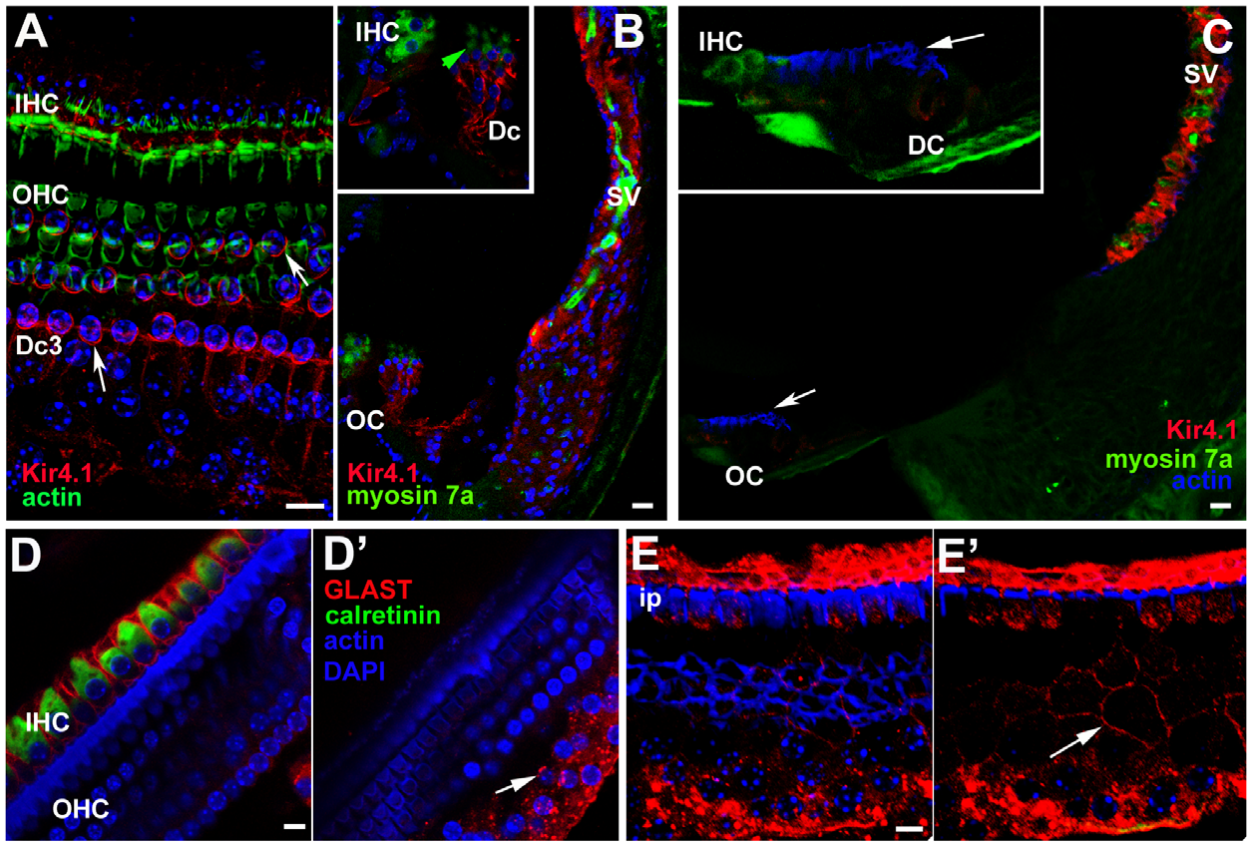
Immunolabelling for Kir4.1 (A–C) and GLAST (D&E). **A,B Kir 4.1 in undamaged organ of Corti.** **A.** Whole mount preparation. Labelling for actin (green) shows the hair cells. Kir 4.1 (red) is present in the Deiters' cell bodies and is strongly expressed in the “cup” region that encloses the base of an OHC (arrows), but there is no labelling in the phalangeal processes (e.g. in the gap between the bodies of OHC2 and OHC3). Kir4.1 is also strongly expressed in the plasma membranes of the supporting cells around the IHC. **B.** Frozen section. The stria vascularis (SV) shows intense labelling (of intermediate cells) with equally intense labelling in the supporting cells of organ of Corti (OC) – shown at higher power in the inset. (Hair cells immunolabelled for myosin 7a (green); green arrow indicates OHC). **C. Kir 4.1 in repaired organ of Corti at 2 weeks post-treatment, C57BL/6 mouse.** With loss of OHC, there is significantly reduced immunolabelling for Kir4.1 in the organ of Corti (OC), shown at higher power in the inset, (arrows indicate actin labelling of the reticular lamina), but Kir4.1 is still strongly expressed in the stria vascularis (sv). **DD'. GLAST in undamaged organ of Corti.** D. GLAST is strongly expressed by supporting cells around the IHC (immunolabelled for calretinin) but is absent from the phalangeal processes of Deiters' cells. D' Confocal optical sectioning at a deeper level shows GLAST labelling at the membranes of the bodies of Deiters' cells (arrow). **EE'. GLAST in repaired organ of Corti in C57BL/6 mouse; 4 weeks post-treatment.** Focus at the plane of the reticular lamina (E) shows the characteristic pattern actin labelling (blue) of cell junctions between Deiters' cells in the repaired epithelium after loss of OHC. GLAST is still strongly expressed by inner phalangeal and inner border cells although IHC have also been lost. Focus at a deeper level (E') reveals labelling for GLAST in the plasma membranes of the Deiters' cells (arrow) delineating their shapes, which show similar irregularities to that revealed by labelling for KCC4 in the repaired epithelium. Scale bars: 10 µm in all panels.

The distribution of labelling for Kir4.1 in the normal tissue differed from that of KCC4. Labelling was confined to the cell body region around the supporting cells' base, and was particularly intense around the “cup” –like formation in which the Deiters' cell encloses the base of the OHC (Fig. 4A,B). However, it was absent from the phalangeal processes. Kir 4.1 labelling was also evident around the IHC. Intense labelling for Kir4.1 was also present around intermediate cells of the stria vascularis along the lateral wall of the scala media (Fig. 4B). With loss of OHC the labelling for Kir 4.1 in Deiters' cells was significantly reduced. In preparations of organ of Corti from which OHC had been lost, examined as whole mounts by confocal microscopy, labelling for Kir 4.1 was barely detectable (not shown). That this was not due to problems with the labelling protocol or the antibody was shown by examination of frozen sections of similar samples in which intense expression in the stria vascularis persisted with essentially no labelling in the organ of Corti (Fig. 4C).

### GLAST

In normal tissue, labelling for the glutamate-aspartate transporter was intense along the plasma membranes of the inner border cells and inner phalangeal cells (Fig. 4D). The pattern of labelling distinguished the outlines of these cell types. Some labelling was also apparent, but weakly, around the Deiters' cell bodies. GLAST labelling was retained in the repaired epithelium after hair cell loss even in regions where there were no IHC (Fig. 4E). Intense labelling around the plasma membranes of inner border and inner phalangeal cells delineated the scar formations at the sites where lost IHC had been replaced by expansion of the surrounding supporting cells. Labelling was also evident amongst Deiters' cells, the labelling around the plasma membranes delineating their shapes (Fig. 4E') in a manner similar to that of labelling for KCC4 in these cells in the repaired epithelium (Fig. 3B').

### Connexin labelling

**Figure 5:**
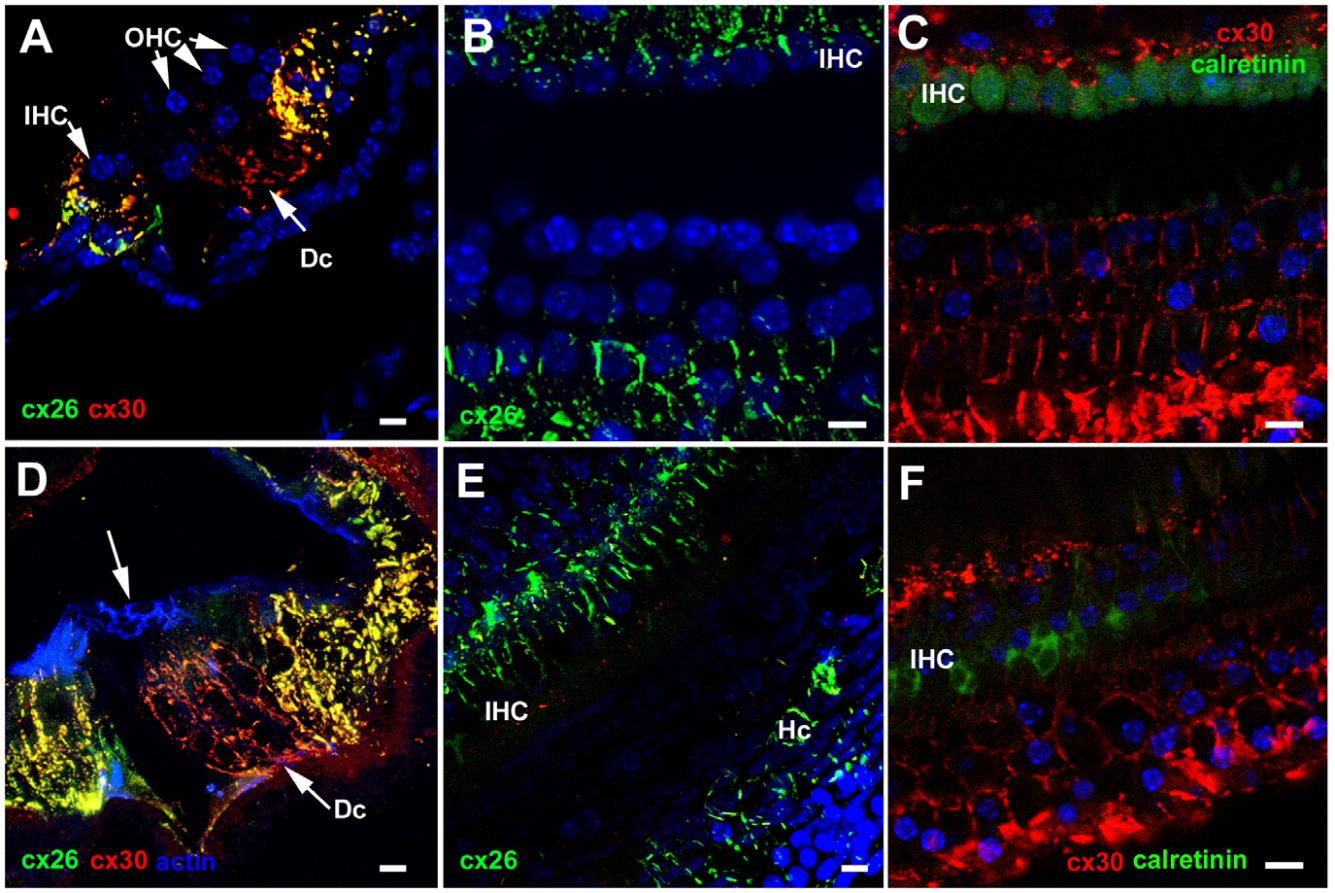
Immunolabelling for Connexin26 and Connexin30. **A–C. Undamaged organ of Corti.** **A. Frozen section.** Cx30 (red) predominates amongst Deiters' cells (Dc), whereas Cx26 labelling (green) is extensively expressed together with Cx30 in cells on the outer (lateral side) of the Deiters' cells (in Hensen's, Claudius and outer sulcus cells) as well as the supporting cells around the IHC and in the inner sulcus. **B. Cx26 labelling in whole mount preparation.** Large gap junction plaques around Hensen's and Claudius' cells, and inner border and inner sulcus cells are labelled, but little labelling around Deiters' cells. **C.Cx30** is present in large gap junction plaques between Deiters' cells. **D–F. Repaired organ of Corti following hair cell loss.** **D. CBA/Ca mouse, 4 days post-treatment.** In a frozen section, Cx30 predominates around Deiters' cells, with extensive Cx26 expression, together with Cx30 in cells to the lateral and medial sides of the repaired organ of Corti. Arrow indicates the characteristics pattern of actin labelling in the reticular lamina of the repaired epithelium. **E. CBA/Ca mouse, 14 weeks post-treatment.** Optical section at approximate level of the cell bodies of Deiters' cells. The pattern of Cx26 labelling is the same as that in the undamaged tissue. **F. C57BL/6 mouse 3 weeks post-treatment.** Cx30 labelling around the bodies of Deiters' cells. The shapes of the cell bodies, defined by the labelling, are much more irregular than those in the undamaged tissue (panel C) but similar to those defined by labelling for KCC4 and GLAST in the repaired epithelium in Figure 4. Scale bars: 10 µm.

In the undamaged epithelium, Cx26 labelling predominated around Hensen's cells and Cx26 and Cx30 were extensively present and co-localised around Claudius' cells as well as around inner border cells and the cells of the inner sulcus (Fig. 5A,B,C). Within the Deiters' cell region Cx30 was present around the cell bodies of Deiters' cells (Fig. 5A,C), but labelling for Cx26 was much less evident in this region (Fig. 5A,B). There was no change in this pattern after loss of all OHC. Cx30 labelling predominated amongst the Deiters' cells whilst labelling for Cx26, along with Cx30, was intense in the regions either side of the organ of Corti strip (Fig. 5D–F). Furthermore, neither successive confocal sections through the depth of the tissue in whole mount preparations nor frozen sections revealed labelling of connexins in the more apical regions of these cells where the expanded phalangeal processes of Deiters' cells came close together, suggesting there was no formation of new gap junctions with the changes in cell-cell contact areas, a conclusion supported from examination of thin sections (Fig. S2). Immunolabelling for Cx43 was undertaken to determine whether this gap junction channel protein was up-regulated. No labelling for Cx43 could be detected in the repaired organ of Corti at any time post-treatment examined (not shown).

**Figure 6:**
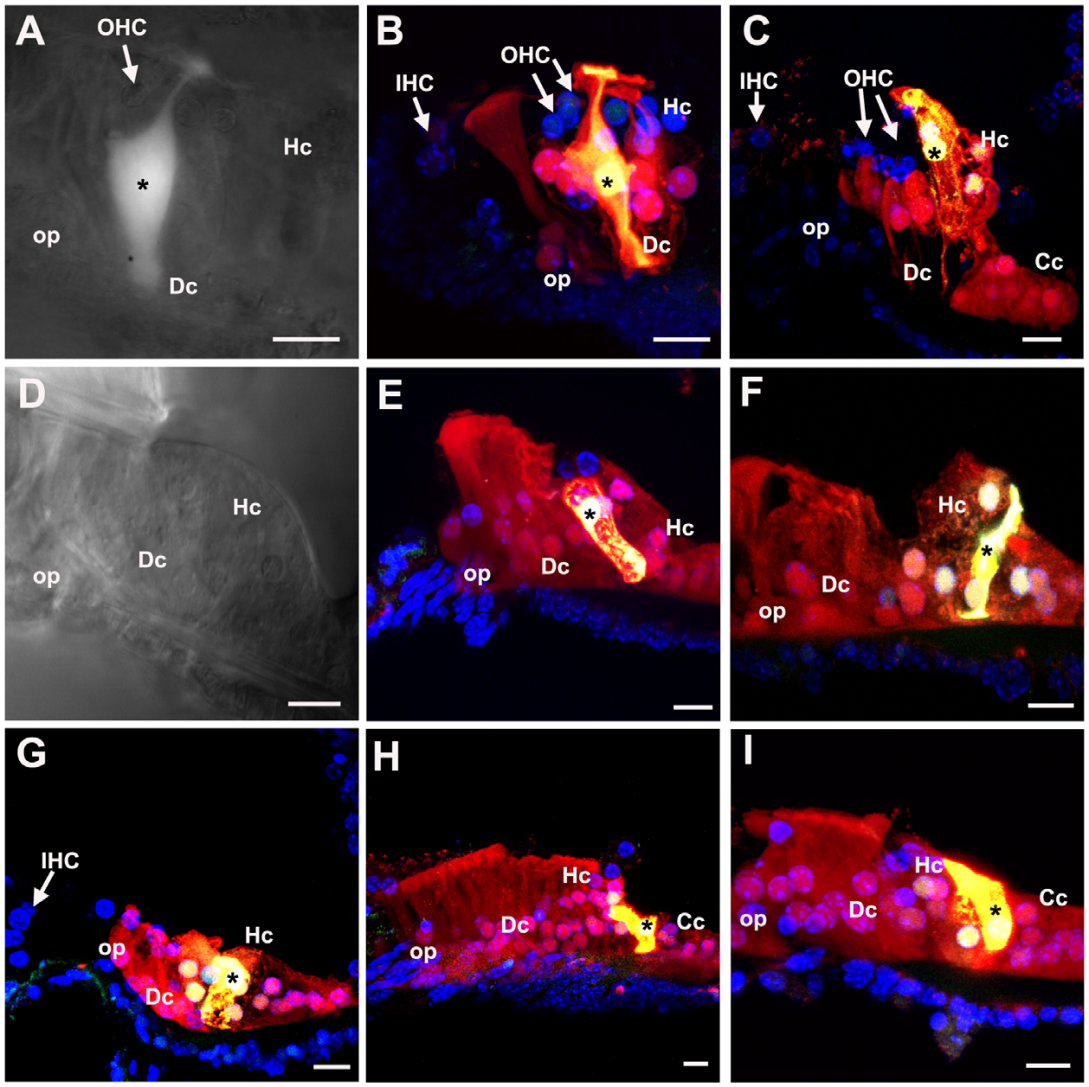
Dye transfer in slice preparations. **A–C Intercellular dye-coupling between supporting cells in untreated P19–P20 mice.** Asterisks denote the injected cell. **A.** Epi-fluorescence videomicrograph of the organ of Corti following a 10-minute whole-cell recording from a Deiters' cell, showing Lucifer yellow fluorescence retained within the single cell. **B.** Post-fixation 30 µm confocal projection through slice shown in A, showing neurobiotin/streptavidin (red) and Lucifer yellow (green) fluorescence. Nuclei were stained with DAPI (blue). Neurobiotin spread from the injected cell to several others within the lateral supporting cell compartment only, and Lucifer yellow was retained within the single Deiters' cell. **C.** Following a whole-cell recording from a Hensen's cell, neurobiotin and Lucifer yellow both spread to several cells within the lateral supporting cell compartment. **D–I. Cochlear supporting cells in kanamycin/bumetanide injected mice retain gap junctional intercellular communication.** Asterisks denote the injected cell. **D.** Infrared differential interference contrast (IR-DIC) videomicrograph showing organ of Corti from a P21 mouse injected with kanamycin/bumetanide at P17. **E–I.** Examples of intercellular dye-coupling between supporting cells in kanamycin/bumetanide treated P21 mice. In all cases there was extensive spread of neurobiotin, and variable spread of Lucifer yellow, in patterns comparable to those in slices from untreated animals. The expansion of Deiters' cells to fill extracellular spaces following loss of OHC is apparent from the dye labelling. Scale bars: 10 µm in all panels.

Pathways of intercellular communication via gap junctions were examined by dye transfer in slice preparations of cochleae of untreated (control) animals at P19 and P20, and in 21 day old mice that had been treated with kanamycin and bumetanide at P17. The patterns of dye transfer in the undamaged fully mature organ of Corti from hearing animals resembled that previously described for rats at the onset of hearing (P12) [34]. When Lucifer Yellow (LY), which can pass through gap channels composed of Cx26 but not those which contain Cx30 [35], together with neurobiotin (NBN), which can pass through gap junction channels of any composition, were co-injected into an individual Deiters' cell LY was retained within the cell whereas NBN spread to other Deiters' cells and outwards to the outer sulcus. It also spread inwards to the outer pillar cell, but it did not pass to the inner pillar cell (Fig. 6A,B). When injected into an individual Hensen's cell, both dyes spread to several neighbouring Hensen's cells but not to Deiters' or Claudius' cells (Fig. 6C). The inability of LY to transfer from the Deiters' cell to its neighbours is consistent with the immunohistochemical labelling patterns showing a predominance of Cx30 but absence (or very low levels) of Cx26 in gap junction plaques between Deiters' cells. Moreover the widespread transfer of NB to the cells on the lateral side of the organ of Corti but its absence from the inner pillar cells and the supporting cells around the IHC confirms in the fully mature tissue what was observed in cochleae from younger animals: that the supporting cells on the lateral side comprise an extensively coupled compartment that is separate from the supporting cells around the IHC, with the border at the level of the pillar cells.

In the organs of Corti of the animals treated with drugs at P17 and then examined at P21, i.e. 4 days post-treatment, all OHC missing and Deiters' cells phalanges had expanded (Fig. 6D), yet dye transfer characteristics were the same as in the control tissue. Following co-injection of the dyes into an individual Deiters' cell, LY was retained in the cell but NBN spread both laterally to Hensen's and Claudius' cells, and medially to other Deiters' cells and the outer pillar cell, but it did not transfer to the inner pillar cell (Fig. 6E). Likewise following co-injection of dyes into a Hensen's cell NBN transferred to other supporting cells up to the outer pillar cell medially and across to the outer sulcus laterally but LY spread only to other Hensen's cells (Fig. 6F–I). Thus, the separate coupled compartments around the OHC and around IHC identified in the normal tissue [34] were retained with hair cell loss. Furthermore the characteristics of the differential dye transfer are consistent with the immunolabelling for connexins in the repaired epithelium that indicate retention of normal patterns of expression.

### Remodelling of the organ of Corti following the initial repair

**Figure 7:**
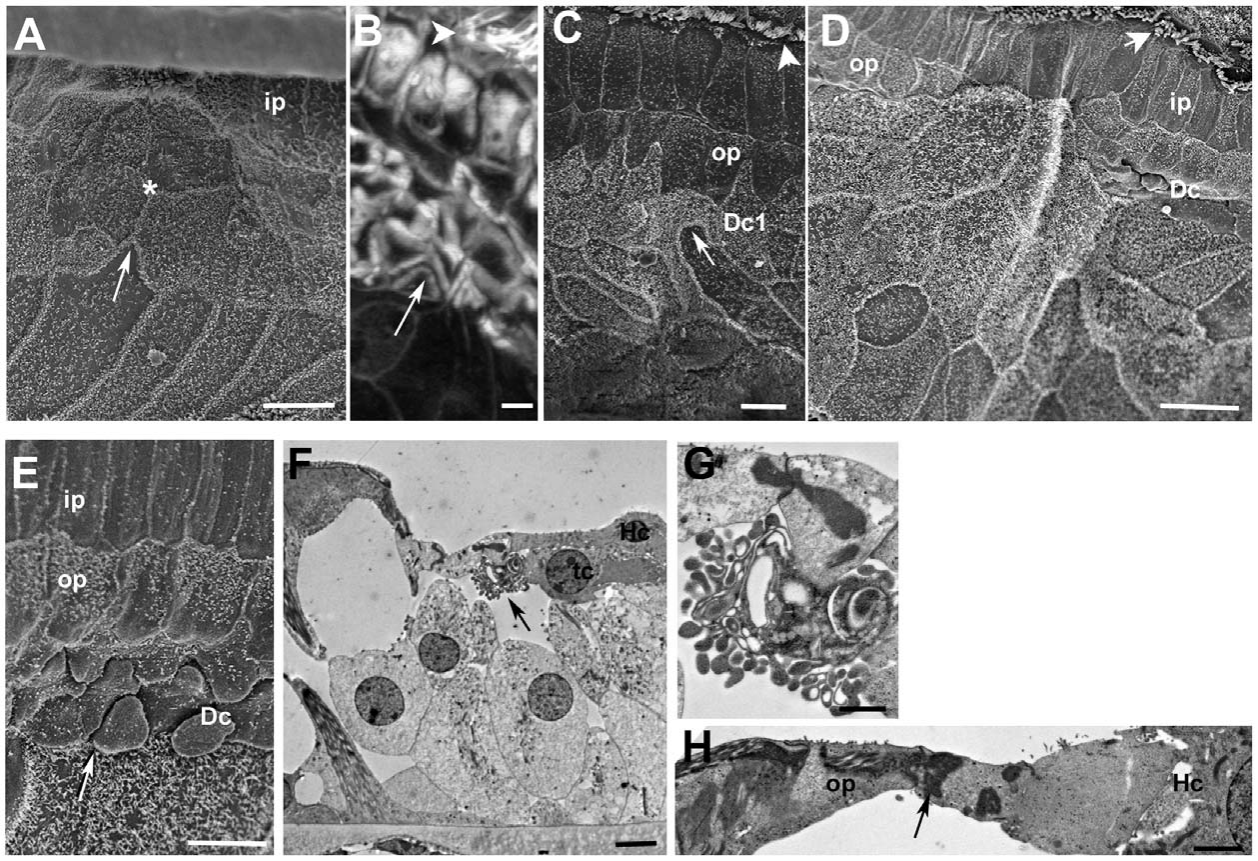
Initial progression of re-modelling of the repaired epithelium. **A. C57BL/6 mouse, 2 weeks post-treatment.** Extension of tectal cell head into the repaired epithelium (arrow). The regular cellular pattern of the repaired reticular lamina is disrupted (asterisk). Scale bar: 5 µm. **B. C57BL/6 mouse 2 weeks post-treatment.** Phalloidin labelling indicates actin assembly at leading edge of the cell advancing into the Deiters' cell region (arrow). IHC are still present (arrowhead). Scale bar; 5 µm. **C. C57BL/6 mouse, 4 weeks post-treatment.** Head of tectal cell expanded (arrow) as far the first row Deiters' cell head. IHC are still present (arrowhead). Scale bar: 5 µm. **D. CBA/Ca mouse, 6 months post-treatment.** Hensen's cells, identifiable by the dense microvilli, cover Deiters'cells (Dc) and contact with the inner pillar cells (ip). IHC are still present (arrowhead). Scale bar: 10 µm. **E. CBA/Ca mouse, 6 months post-treatment.** The apical heads of the Deiters' cells (Dc) appear to be extruding from the apical surface (arrow). Scale bar: 5 µm. **F. C57BL/6 mouse; 13days post-treatment.** Hensen's cell (Hc) extends towards the pillar cell region. The third row Deiters' cell (arrow) appears to be fragmented; its basal aspect show features of cellular degeneration (shown at higher power in panel G). The bodies of the Deiters' cells within the corpus of the epithelium appear intact with prominent microtubule bundles. Scale bar: 5 µm. **G.** The apical fragment of Deiters' cell indicated by the arrow in F. The cytoplasmic continuity of the most apical junctional region with more basal denser region enclosed within convoluted plasma membrane is apparent. Series of thin sections through this structure confirmed this as a fragment of one cell. Scale bar: 1 µm. **H.** Section through the reticular lamina in a region where Hensen's cell is approaching the head of an outer pillar cell (op). The head of the outer pillar cell is recognised by the microtubule bundles. Only a single microfilament assembly (arrow) characteristic of the junction of a Deiters' cell is evident, suggesting withdrawal, or loss, of Deiters' cell heads from the apical surface of the epithelium (compare with Figure 1E). Scale bar: 2 µm.

**Figure 8:**
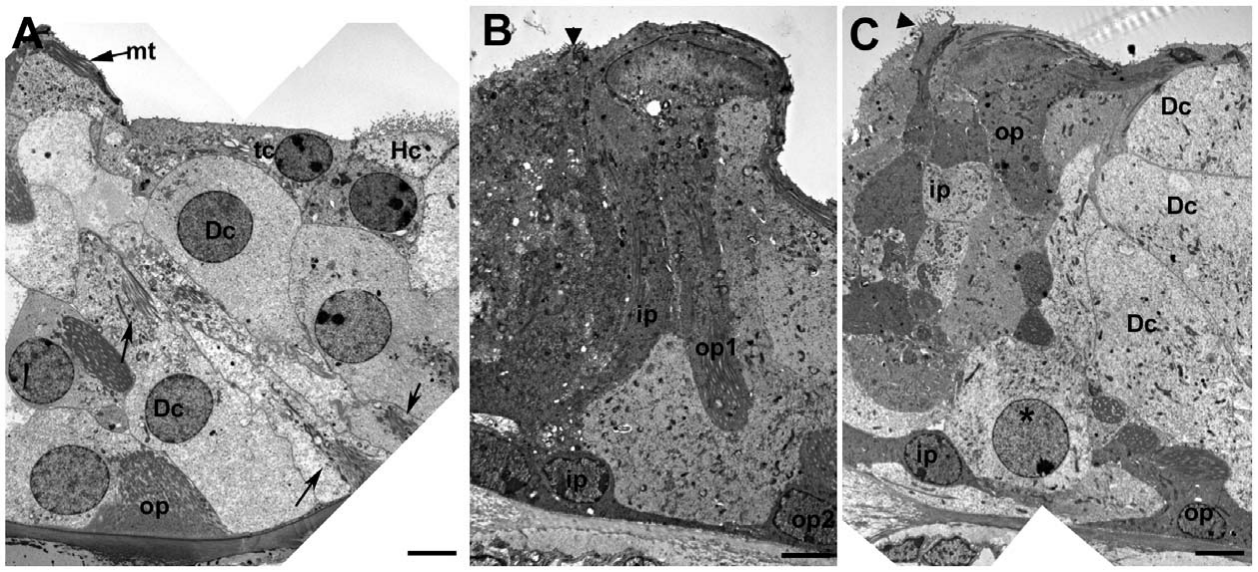
Deiters' cell migration. **A.** Tectal cell (tc, dense cytoplasm) followed by Hensen's cell (Hc, microvilli at the surface) extends across the Deiters' cell to contact the head of the outer pillar (recognised by the microtubule bundles parallel to luminal surface, mt). The Deiters' cell bodies (Dc) fill the entire epithelium up to the outer pillar cell phalangeal processes, but retain their cytoplasmic characteristics, including organised microtubule bundles at the cell base and within the cell body (arrows), but they appear to be entirely enclosed within the epithelium with no exposure to the apical surface. Scale bar: 5 µm. **B** and **C.** Cells filling the tunnel of Corti in repaired epithelium with intact pillar cells. In B, the angle of section has revealed the phalangeal process of one outer pillar cell (op1) and the cell body of its neighbour (op2). A cell is filling the tunnel of Corti and spreads to the outer side through the gap that normally exists between adjacent outer pillar cells. Its cytoplasmic characteristics are consistent with those of a Deiters' cell. In C, the epithelium is cut at a larger angle relative to the perpendicular through the epithelium to reveal several outer and inner pillar cells. The cell (asterisk in nucleus) occupying the space between the inner and outer pillar shows the charactersitics of a normal Deiters' cell (Dc). Its nucleus appears normal with no evident apoptotic or necrotic features. In both B and C, IHC are lost and expansion of the inner border and inner phalangeal cells has closed the lesion (arrowhead). Scale bars: 5 µm.

**Figure 9:**
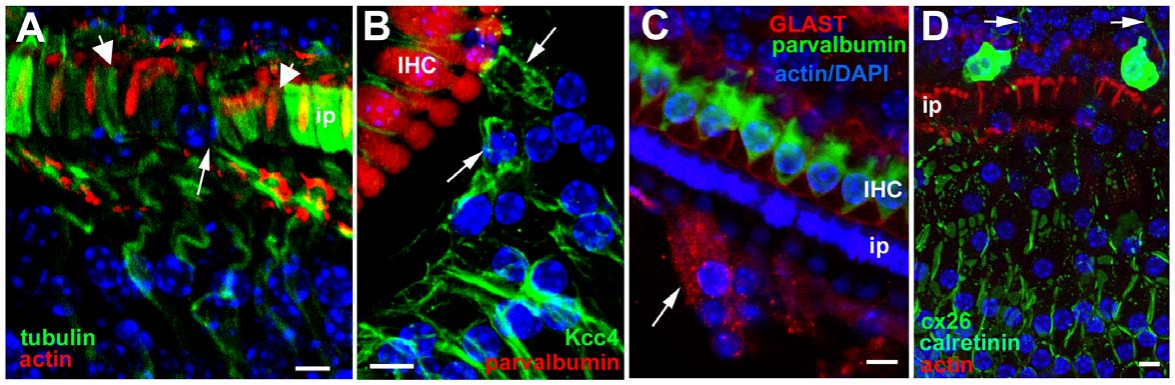
Immunolabelling following the progressive re-modelling of the repaired epithelium. **A.** Immunolabelling for acetylated tubulin (green) reveals microtubules in cells across the Deiters' cell region approaching the inner pillar cells. At the arrow, a nucleus in the position of the tunnel of Corti is associated with microtubules extending back into the Deiters' cell region. The heads of some inner pillar cells (ip) are intensely labelled for acetylated tubulin whereas in others the labelling is significantly reduced or absent (larger arrows) suggesting depolymerisation of the microtubule bundles in pillar cell heads. **B.** Labelling for KCC4 (green) is apparent in cells in the location of, and entering, the tunnel of Corti (arrows) Inner hair cells are labelled for calretinin (red). **C.** Cell labelled for GLAST (arrow) in the location of the tunnel of Corti. **D.** Labelling for Cx26. Large plaques of labelling around the borders of cells across the entire outer side of the organ of Corti up to the level of the inner pillar cells. The pattern of labelling is consistent with that of Hensen's and Claudius' cells. Some inner hair cells labelled for calretenin survive and these cells retain innervation (arrows), also immunolabelled by antibodies to calretinin. Scale bars: 5 µm.

In the repaired epithelium, following hair cell loss, a progressive remodelling of the tissue occurred such that short segments of organ of Corti containing no recognisable Deiters' cells were bordered either side by regions with obvious Deiters' cells. The initiation of the re-modelling was seen as an extension of the apical head of an individual tectal cell that expanded into and displaced third row Deiters' cells (Fig. 7A,B,C). Phalloidin labelling suggested the presence of actin accumulations at the leading edge of such cells (Fig. 7B). The advance of these cells into the Deiters' cell region disrupted the regular arrangement of cell apices within the repaired epithelium (Fig. 7A,B,C). Cells with large surface areas and with apical surface characteristics of Hensen's cells, followed by cells with features similar to those of the normal Hensen's and Claudius' cells neighbours, came to cover the entire surface of the organ of Corti up to the level of the surface of the inner pillar cells (Fig. 7D). Disruption of the Deiters' cells at their apical surface occurred as invading cells advanced towards the pillar cells. The heads of Deiters' cell sometimes appeared to be in the process of being extruded from the epithelium (Fig. 7E); and thin sections showed that in regions where Hensen's cells were approaching the pillar cells, there were fragments of Deiters' cells, separated from intact cell bodies within the corpus of the epithelium, that were retained at the apical surface with apparently intact junctions with the neighbouring cells, but showing features suggestive of degeneration at their basal aspect (Fig. 7 F,G). Examination of serial sections confirmed that the morphology shown in Fig. 7G was that of the apical fragment of a Deiters' cell and not engulfment by a phagocyte. At the apical surface of the epithelium where the Hensen's cells appeared to be closing in on the pillar cells, the number of intercellular junctions across the Deiters' cell region was reduced: those junctions with which thick microfilament assemblies characteristic of the tight-adherens junctions of Deiters' cells disappeared (Fig. 7H), consistent with withdrawal of Dieters' cells from the apical surface. However, there was no evidence from either SEM or thin sections of significant lesions through the apical surface of the epithelium as Deiters' cells came to be covered over, and within the epithelium these cells retained distinct morphological characteristics with seemingly intact microtubule bundles (Fig. 8A). Thin sections also revealed the tunnel of Corti to become completely filled with cells while the phalangeal processes of both outer and inner pillar cells remained erect with intact bundles of microtubules (Fig. 8B,C). The cytoplasmic characteristics of the cells occupying the tunnel of Corti indicated that they were Deiters' cells. In wholemount preparations of repairing tissue, nuclei were present directly below the heads of the pillar cell where normally no cell nuclei are located and cells in this position showed labelling for acetylated tubulin (Fig. 9A), KCC4 (Fig. 9B) and GLAST (Fig. 9C) supporting the contention that Deiters' cells spread into this region. The nuclei of such cells and those of the cells occupying the tunnel of Corti as seen in thin sections (Fig. 8C) were of normal appearance, with no obvious apoptotic or necrotic features, indicating the cells filling the tunnel of Corti were viable.

**Figure 10:**
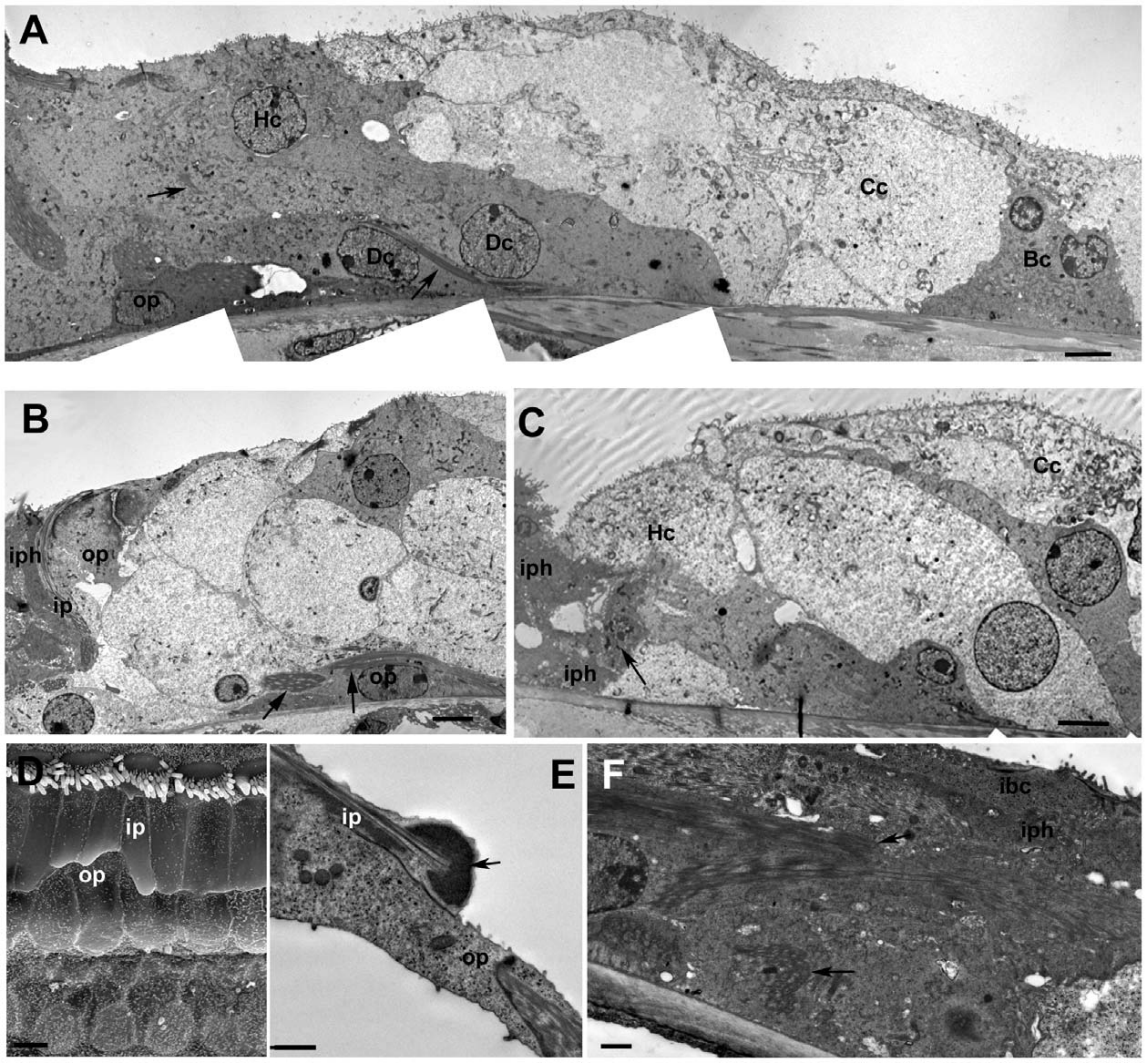
A–C. Further progression of re-modelling on the lateral side of the pillar cell region. **A.** The outer (lateral) side of the organ of Corti during re-modelling to show relative positions of different cell types. Boettcher's cells (Bc) are in position at the lateralmost side and appear to form the outer border. Deiters' cells (Dc) are flattened and covered by Claudius' and Hensen's cells (Cc, Hc) that have expanded medially (towards the pillar cells), but they retain their specialised microtubule bundles (arrows). Scale bar: 5 µm. **B.** The body of the outer pillar (op) is flattened and covered by Deiters' cells, but prominent, organised microtubule bundles are retained (arrows). The head of the outer pillar appears to have separated from the cell body as Deiters' cell spreads through to the tunnel of Corti. Scale bar: 5 µm. **C.** Cell with numerous microvilli at the apical surface and the cytoplasmic characteristics of Hensen's cell (Hc) in contact with inner phalangeal cell (identified by the dense cytoplasm) at the approximate site where the IHC used to be. A cell with cytoplasmic characteristics of Claudius' cell (Cc) expands across the apical surface of the epithelium. One inner phalangeal cell encloses cell debris (arrow). Scale bar: 5 µm. **D–F. Inner pillar cell during remodelling.** **D.** Heads of inner pillar cells (ip) retract to expose the apical surfaces of the outer pillar cells beneath. Scale bar: 5 µm. **E.** The retracting tip of the head of the inner pillar cell is associated with a sub-membrane density that resembles the thin section appearance of a microfilament assembly. Scale bar: 2 µm. **F.** The inner pillar cell becomes flattened and covered by the expanding inner phalangeal and inner border cells but it retains prominent organised microtubule bundles (arrows). Scale bar: 2 µm.

In immunolabelled whole mount preparations, labelling for Cx26 was intense at the borders between cells delineating large gap junction plaques across the Deiters' cell region, where normally Cx26 does not predominate, up to the level of the inner pillar cells (Fig. 9D). This labelling pattern is consistent with identifying the cells “invading” the organ of Corti as originating from the population of cells at the outer edge of the normal organ of Corti: Hensen's and Claudius cells. The organ of Corti on the lateral side was significantly reduced in height and became a “solid” tissue upon the basilar membrane with no extracellular spaces present. Cells with cytoplasmic characteristics of Hensens' and Claudius cells covered the apical surface of the epithelium, but Boettcher's cells remained distinct and in place (Fig. 10A). Both outer and inner pillar cells remained erect with prominent bundles of parallel microtubules and actin assemblies at the base of the cells, for some time. With expansion and apparent migration of Deiters' cells inwards the phalangeal processes of outer pillar cells collapsed inwards (i.e. towards the modiolus, the direction in which the cells from outside the organ of Corti strip appeared to be progressing), and there was evidence that the apical head region became detached from the phalangeal process but microtubule bundles were still prominent within the cell body region which became covered with Deiters' cells (Fig. 10B).

**Figure 11:**
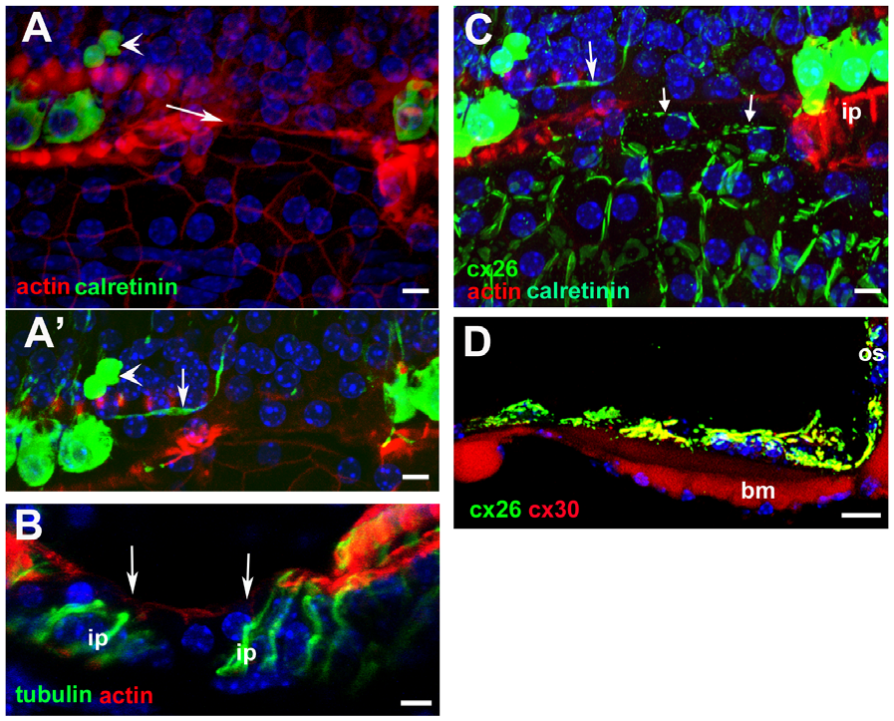
Characteristics of the flattened epithelium. **A.A'** Confocal optical sections at different depths of a whole mount preparation. Actin labelling in a delineates the borders of cells forming a continuous sheet that interrupts the strip of repaired epithelium. A thicker actin band (arrow in A) is present delineating the site at which the cells encroaching from the outer side meet the cells in the position of the inner phalangeal cells. IHC, labelled for calretinin, are still present either side of the patch of squamous-like epithelium. Clumps of calretinin labelling, indicative of degenerating IHC, are located in the approximate position of inner border cells (large arrows in A and A'). In A' calretinin labelled afferent nerve dendrite in the region devoid of IHC appears to have become re-directed to run longitudinally along the organ of Corti towards a remaining IHC. **B.** A squamous-like patch (between arrows) is bordered either side by inner pillar cells (ip) identified by intense labelling for acetylated tubulin. There is no labelling for acetylated tubulin in the cells of the “flat” epithelium and no indication of cells with reduced labelling for acetylated tubulin that could be indicative of transition from specialised supporting cells to non-specialised cells. **C.** Same whole mount preparation as shown in A (and A') after demounting then additionally re-labelled for Cx26. Cx26 labelling reveals large plaques between all the cells that constitute the squamous-like epithelium. The small arrows indicates Cx26 labelled plaques at the border between the squamous-like cells migrating from the outer side and the cells that have replaced the lost IHC. **D.** Frozen section through a region of squamous-like epithelium double-labelled for Cx26 (green) and Cx30 (red). Cx26 and Cx30 are co-expressed (appearing yellow) by most of the cells across the entire basilar membrane (bm), a labelling pattern consistent with that of Claudius' cells in the undamaged organ of Corti. Scale bars: 5 µm in all panels.

In many cases loss of IHC was often not evident until the re-modelling of the lateral side of the organ of Corti was well advanced and Hensen's cells had reached to the level of the inner pillar cells. With death of IHC, the inner border cell expanded to cover the lesion and with collapse of the outer and inner pillar cells, those cells advancing from the outer edge of the organ of Corti came to contact the inner phalangeal cells on inner edge (Fig. 10C). A prominent band of actin labelling ran across the line of contact between these inner and outer domains at the approximate site where the junction between the inner pillar cell and the IHC was previously located (Fig. 11A). At such stages cell debris was evident inside the inner phalangeal cell (arrow in Fig. 10C), and immunolabelling for calretinin, which labels inner hair cells, showed clumps of labelled material at the approximate location of the cell bodies of inner phalangeal or inner border cells (Fig. 11A,A'), suggesting that one or other or both of these cell types may take up the debris of dying IHC, similar to the removal of the debris of dying OHC by Deiters' cells [40], [48].

Remodelling of inner pillar cells was evident from quite early in the re-organisation. Often where outer pillar cells were still intact and erect, the heads of the inner pillar cells were seen to retract to uncover the heads of outer pillar cells (Fig. 10D). The retraction appeared to involve accumulation of microfilaments at the distal retracting tip (Fig. 10E) and the depolymerisation of the microtubules (Fig. 9A). However, the inner pillar cell phalanges remained erect at least until after collapse of the outer pillar cell (Fig. 10B). The phalangeal processes of the inner pillar cells eventually collapsed outwards (i.e. in the direction opposite to that to which the outer pillar cells collapsed) as the cells on the inner side of the normal of Corti strip expanded outwards. However, even when reduced in height to that of the cuboidal-like cells that now covered the basilar membrane, the presence of organised bundles of microtubules seen in thin sections, indicated that specialisations of inner pillar cells were retained (Fig. 10F, 11B).

**Figure 12:**
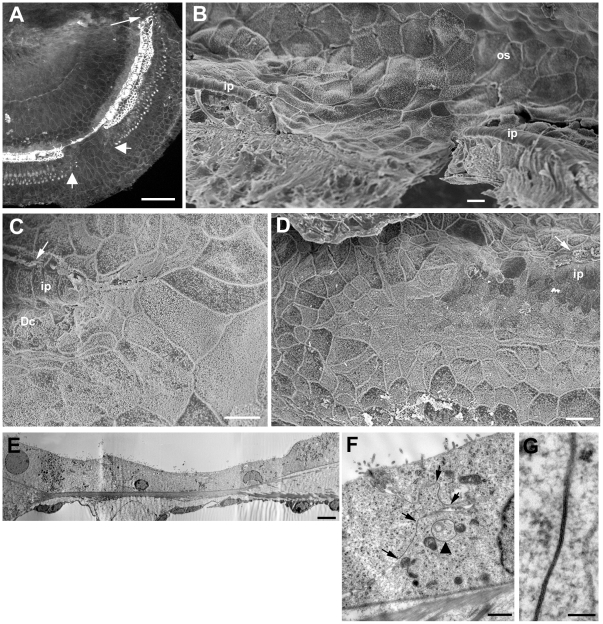
Morphological characteristics of the squamous-like epithelium. **A. C57BL/6 mouse at 6 weeks post-treatment.** Actin labelling in whole mount preparation of the apical coil. The arrow indicates the apical tip of the organ of Corti. A patch of the flat epithelium, indicated by the larger arrows, interrupts the strip of repaired epithelium. Scale bar: 100 µm. **B. C57BL/6 mouse at 4 weeks post-treatment.** Mid-basal region of the cochlea viewed towards the lateral wall (from inside outwards). Pillar cells (ip) are still erect either side of a patch of flattened epithelium. The sheet of flattened epithelial cells is continuous with the cells of the outer sulcus (os). Scale bar: 10 µm. **C.** Edge of squamous patch where it cuts across the repaired epithelium. The enlarged surfaces of cells that normally reside on the outerside of Deiters' cells curve round the end of the strip containing Deiters' cells (Dc) and pillar cells (ip). IHC still present (arrow). Scale bar: 10 µm. **D.** The apicalmost tip of the organ of Corti. The surfaces of cells either side of the strip of cells containing Deiters' and pillar cells, curve across the termination point of that strip in a similar pattern to that at the edges of the patches of squamous-like epithelium. The surface features of those cells are also similar to those that create the flat epithelium. Scale bar: 10 µm. **E.** Thin section through a region of flattened epithelium. The cells across the basilar membrane are all similar in morphology and show relatively few organelles or other cytoplasmic specialisations. Their features are reminiscent of those of Claudius' cells. Scale bar: 2 µm. **F** and **G.** Details of cell forming the flat epithelium. The cell cytoplasm is relatively unstructured and there are few organelles. Large gap junction plaques occupy much of the plasma membrane along the contact between adjacent cells. The extent of two gap junctions are defined by the pairs of arrows in panel F, the lower contact region viewed at higher power showing the characteristic thin section morphology of a gap junction. Annular gap junctions are also evident in the cell cytoplasm (arrow in panel F), suggesting continuing turnover of gap junction plaques. Scale bars: 1 µm in F; 0.1 µm in G.

The contact between the two domains of supporting cells resulted in the creation of a single continuous layer of cells across the entire width of the basilar membrane. This developed in patches dispersed along the length of the organ of Corti, in apical (Fig. 12A) as well as basal (Fig. 12B) coils rather than developing and enlarging along the length of the basilar membrane from one initial site of formation. Patches of this simple epithelium were apparent in regions where IHC were still present, with IHC evident on both the apical and basal sides of the patch (Fig. 11A, C; Fig. 12B,C), as well as in regions where all IHC had been lost but pillar and Deiters' cells were still present in a “repaired” epithelium on both edges of the patches (Fig. 12A). However, in other regions where all IHC and OHC were absent, there was no evidence of tissue re-organisation (Fig. 2B, G). These variabilities in the cellular environment in which re-modelling occurred were evident in both of the mouse strains examined. At the interface between the non-specialised epithelium and the repaired organ of Corti where recognisable pillar and Deiters' cells remained, the cells across the strip of repaired organ of Corti followed a curve running from behind the third row of Deiters' cells across the inner pillar cells to meet the apices of inner border cells (Fig. 12C). These non-specialised epithelial regions have a similar appearance to the termination point of the normal organ of Corti at the extreme apical tip of the spiral (Fig. 12D).

The cells forming the simple epithelium were thin, 5–10 µm tall (Fig. 12E), with large, irregular surface areas organised in a pavement pattern reminiscent of squamous epithelial tissue (Fig. 11A,C; Fig. 12B,C). Their cytoplasm was relatively electron luscent and they possessed relatively few organelles (Fig. 12E, F). Labelling for connexins revealed large gap junction plaques and intense expression of both Cx26 (Fig. 11C) and Cx30 across the entire basilar membrane, often apparently co-localised (Fig. 11,D). Connexin labelled plaques were also evident between the cells at the junction where the cells covering the outer part of the basilar membrane met those on the inner side at the approximate site where the inner hair cells were once located (Fig. 11C). Thin sections confirmed the presence of large gap junctions between adjacent cells occupying a significant proportion of their lateral plasma membranes (Fig. 12F,G). Large annular gap-junctions [33] also were present inside many cells (Fig. 12F), suggesting continuing turnover of gap junction plaques. There was however, no labelling for Cx43 (not shown). There was also no labelling for acetylated tubulin (Fig. 11B) or KCC4 within the regions of squamous-like epithelium. The characteristics of the cells forming the simple epithelium are consistent with those of Claudius and Hensen's cells covering the basilar membrane and coming into contact with inner sulcus cells. The number of nuclei across the basilar membrane from the outer sulcus to the inner edge of the inner sulcus was counted in sections to assess the number of cells forming the flat epithelium in comparison with the repaired epithelium with intact Deiters' and pillar cells. In regions of simple epithelium there were markedly fewer cells: 10±1.7 (n = 25 samples) versus 17.5±1.6 (n = 29).

### Afferent innervation to IHC

Antibodies to calretenin provide labelling not only for IHC, but also for their afferent innervation. Where IHC persisted in regions where re-organisation was on going, the afferent dendrites running radially in the medial direction away from the IHC were labelled (Figs. 9D; 11A') indicating persistence of the innervation where the IHC remained. At patches where the squamous-like epithelium covered the entire basilar membrane, most dendrites across the region where IHC were absent were lost but a few sometimes remained and some of these appeared to have changed direction to run longitudinally along the organ of Corti towards the remaining IHC (Fig. 11A'). Whether these formed functional synaptic contacts was not examined.

### Genetic background and rate of reorganisation

The characteristics of progressive re-organisation were similar in both of the mouse strains examined. However, although almost complete loss of all OHC occurred in both strains of mice within 48 hours of the drug treatment, the rate at which re-modelling occurred was markedly different. In the C57BL/6 mice, evidence of re-modelling was sometimes present by 2 weeks post-treatment and patches of squamous-like epithelium were consistently present by 3–4 weeks. In the CBA/Ca mice, the repaired organ of Corti formed of expanded Deiters' cells persisted along the entire organ of Corti spiral for prolonged periods and initiation of re-modelling was not seen until 4–6 months after the initial OHC loss. In both strains of mice, however, re-modelling could be initiated while IHC persisted in the epithelium (e.g. Fig. 7C for C57Bl6; 7D for CBA/Ca) whereas, also in both strains, there could be complete loss of all IHC as well as all OHC without initiation of migration of Hensen's cells (e.g Fig. 2F for CBA/Ca; 2G for C57BL/6). This suggests loss of IHC is not a trigger for initiation of the re-organisation.

### Macrophages

In several other tissues where re-modelling occurs after injury, macrophages are attracted to the sites of damage. They phagocytose and clear the debris of dying cells, and release cytokines and other factors that regulate tissue repair and wound healing. We examined for the presence of macrophages to determine whether they are likely to play a role in the repair of the organ of Corti following hair cell loss.

**Figure 13:**
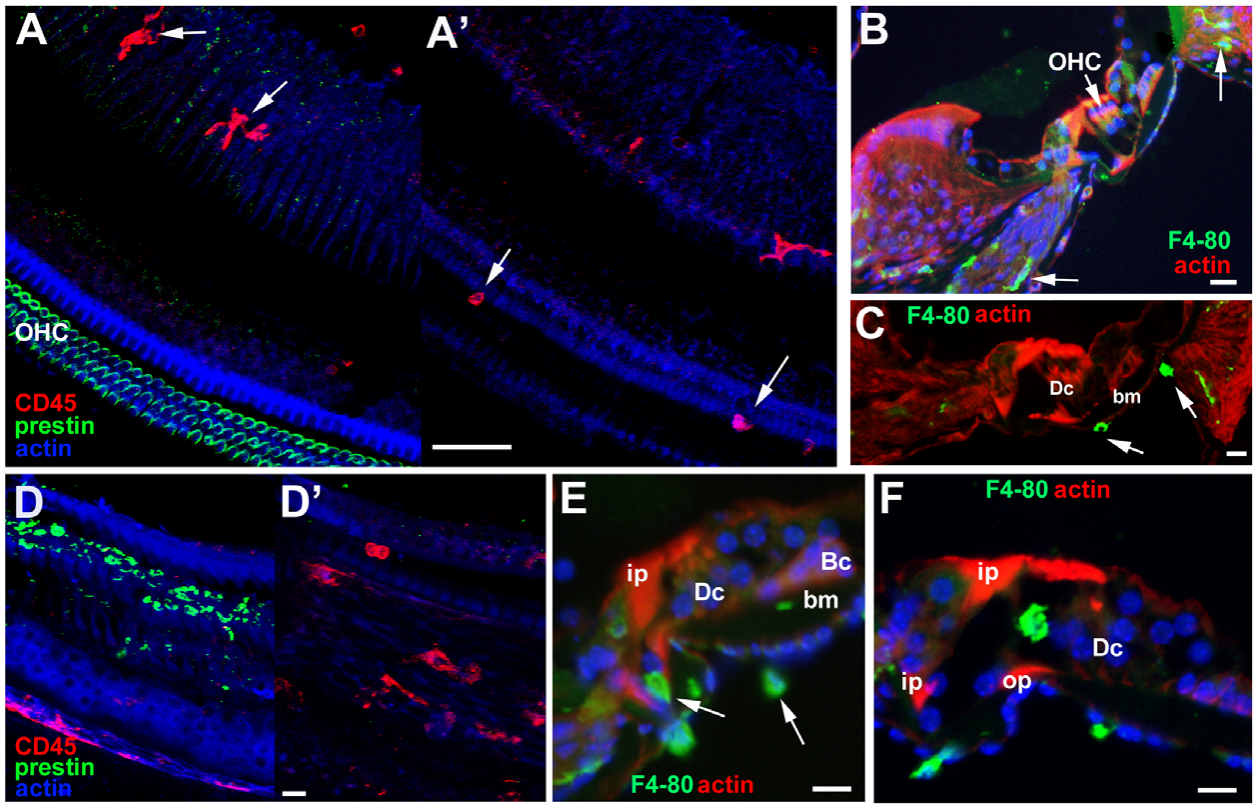
Immunolabelling of Macrophages. **A–C Undamaged organ of Corti.** **A A'.** Immunolabelling for CD45 (red) and for prestin (green) to mark OHC. Focus at the level of the body of the organ of Corti in A and the basilar membrane in A'. In A, macrophages in the nerve tract but none within the organ of Corti itself. Macrophages on the underside of the basilar membrane in A'. Scale bar: 50 µm. **B,C.** F4-80 in frozen sections. B. Macrophages (indicated by arrows) within the ligament of the lateral wall and in the nerve tract (arrows). C. Macrophages (arrows) on the underside of the basilar membrane. Scale bars: 20 µm. **D–F. Following hair cell damage.** **D D'. C57BL/6 at 24 hours post treatment.** D. Focus at the level of the body the organ of Corti shows debris of degenerating OHC labelled for prestin (green). There are no cells labelled with macrophage marker CD45 at this level indicating they are absent from the body of the organ of Corti at the time when hair cell degeneration is occurring. D'. Focus at the level below the organ of Corti reveals CD45 positive cells (red), remain on the underside of the basilar membrane. Scale bar: 20 µm. **E. CBA/Ca mouse; 48 hours post treatment.** Frozen section labelled for cells expressing F4-80 (green). Macrophages are present on the underside of the basilar membrane and in the nerve tract (arrows) but are absent from the body of the organ of Corti at a time when OHC loss is on-going. Scale bar: 10 µm. **F. CBA/Ca mouse 7 days post-treatment.** Frozen section labelled for cells expressing F4-80. All OHC have been lost and all debris cleared by this time. Large cell of irregular shape expressing the macrophage marker F4-80 (green) is within the tunnel of Corti. Scale bar: 10 um.

In the undamaged organ of Corti cells with the morphological appearance of macrophages (in thin sections, not shown), or cells which were immunolabelled in whole mount preparations or frozen sections for CD45 (Fig. 13A) or the mouse macrophage marker F4-80 (Fig. 13B,C) were apparent in the lateral wall, the spiral ganglion and in the nerve tract running up towards the habenula perforata (Fig. 13A, B) (the point through which nerves pass into the organ of Corti). They were also evident on the underside (scala tympani side) of the basilar membrane (Fig. 13B',D), but no macrophages were detected within the body of the organ of Corti itself. There was also no evidence of macrophages within the body of the organ of Corti during the early period post treatment (24–48 hours) when extensive death of OHC was rapidly progressing (Fig. 13D,E), and there appeared to be no significant increase in the numbers of macrophages at this time in comparison with undamaged tissue. This suggests that death of hair cells does not promote significant recruitment of macrophages directly to the sites of damage and is consistent with evidence for the important role of phagocytic activity of Deiters' cells in clearing much of the debris of dying OHC [40], [48].

**Figure 14:**
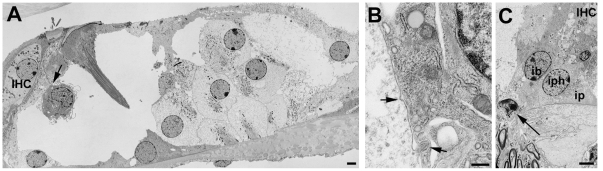
Macrophages in thin sections. **A. CBA/Ca mouse 4 weeks post-treatment.** All OHC lost, debris cleared and Deiters' cell expanded. A cell with morphological characteristics of a macrophage in the tunnel of Corti closely abuts the inner pillar cell. The region of close contact indicated by the arrow is shown at higher power in panel B. Scale bar: 2 µm. **B.** Detail of the contact region between the macrophage and the inner pillar cell. The plasma membranes of the cells are closely apposed and parallel to each other. Endocytotic opening on the inner pillar side (upper arrow) and protrusion from inner pillar cell enclosed by macrophage (lower arrow) indicate activity reminiscent of an immune synapse between the cells. Scale bar: 0.5 µm. **C.** Region of the habenula perforata – the opening through the bony lip by which neural dendrites enter the organ of Corti - below the inner phalangeal cells (iph) and IHC. A cell which is pleomorphic in shape with an irregularly shaped nucleus, characteristics of macrophages, is present within the habenula (arrow). This may indicate that macrophages can gain access to the organ of Corti along the nerve tract. Scale bar: 2 µm.

Macrophages within the organ of Corti were more prominent, in regions of complete OHC loss at later times (from about 7 days post-treatment), after all hair cell debris had been cleared. Cells that labelled for F4-80 (Fig. 13F) with the morphological appearance of macrophages (Fig. 14A), were observed within the tunnel of Corti. Macrophages extended projections that contacted the phalangeal processes of one or other of the pillar cells and their bodies came into close contact with the phalangeal processes (Fig. 14A). Along this point of contact, the plasma membranes of the two cells were closely parallel and vesicles opening both from the pillar cell and from the macrophage into the gap between the cells were evident (Fig. 14B), suggesting the formation of active synapses between the macrophage and the pillar cell. At times when macrophages were present within the organ of Corti, the number of F4-80 positive cells and cells with morphological features of macrophages in the nerve tract and close to the habenula perforata (Fig. 14C) appeared to be greater than in undamaged tissue. This might suggest that macrophages gained entry to the body of the organ of Corti through the habenula perforata but the numbers of labelled/macrophage-like cells in this region was not quantified.

Macrophages were only evident, however, within the repaired organ of Corti, with erect pillar cells and Deiters' cells in their correct positions, before any obvious re-organisation of the epithelium had begun but their occurrence within the body of the organ of Corti was uncommon. In thin sections and frozen sections that included the whole cochlear height, and in which sections were taken at several depths through the tissue, to enable an assessment of the entire cochlea, only in approximately 10–15% of the cochleae were macrophages evident within the body of the organ of Corti. Even in those, only 1–2 macrophages in that location were observed in an individual cochlea. Similarly small numbers of macrophages within the organ of Corti were observed in whole mount preparations where they could also be identified from their plieomorphic nuclei stained with DAPI and thus potentially identifiable in most whole mount samples examined by confocal microscopy. Macrophages were never identified in areas where re-organisation towards the establishment of the squamous-like epithelium was progressing, nor in association with that “flat” epithelium itself in either thin sections, frozen sections, or whole mount preparations. This relative rarity of macrophages within the organ of Corti, suggests they are not specifically recruited to the repairing tissue.

## Discussion

### Characteristics of the repaired organ of Corti

Of the functional specialisations of supporting cells explored in this study, only one, Kir4.1, appeared to be lost following loss of OHC. Unlike KCC4 which is normally distributed along the plasma membrane both of the cell body and the phalangeal processes of the Deiters' cells, Kir4.1 distribution is restricted. It is expressed in the plasma membrane around the cell body and particularly intensely in the cup region that encloses the base of an OHC and its nerve endings, but it is not expressed in the phalangeal processes. We have confirmed this distribution in immunogold-labelled preparations of the mouse and guinea pig organ of Corti (Jagger and Forge, unpublished). This suggests polarized distribution of Kir4.1 implying particular trafficking pathways to specific locations and the maintenance of membrane domains that accommodate the protein. With loss of hair cells, the supporting cells are stimulated to change shape. It is possible that the failure to detect Kir4.1 in Deiters' cell after hair cell loss is a consequence of a re-distribution of the protein throughout the entire plasma membrane and its “dilution” concomitant with the cell shape change. However, this seems unlikely. The protein appeared to be lost entirely even from the cell body region where shape change initially is less pronounced. Moreover, the antibody used was apparently quite sensitive given the intense labeling that persisted in the lateral wall. Although an analysis by PCR is perhaps required to confirm, it seems reasonable to conclude that the immunolabelling findings reported here do reflect a down-regulation of Kir4.1.

It has been suggested that down-regulation of Kir4.1 might be an indicator of a potential to re-enter the cell cycle since this has been found to be the case in Muller glia cells of the retina [49]. Supporting cells have been considered to be glial-like cells and thus similar to Muller cells. The down-regulation of Kir4.1 appeared to be quite an early event following loss of hair cells and at similar stages there was some evidence of a small number of Deiters' cells losing contact with the underlying basilar membrane and their nuclei moving to a more apical location in the cell, features that can presage cell division. However, there is no other indicator of de-differentiation and there is no evidence that supporting cells re-enter the cell cycle. The loss of Kir4.1 would seem more probably related to changes in cellular morphology and membrane re-organisation and thus that loss of Kir4.1 is related to a change in cellular physiology rather than an indication of de-differentiation.

Although Kir4.1was lost, which would presumably affect K^+^ uptake, intercellular communication pathways via gap junctions between supporting cells were apparently unaffected. One suggested role for gap junction mediated communication between supporting cells, by which functional syncitia are created [34], is to provide a means for the intracellular passage of K^+^ out of the sensory epithelium [50], [51]. The patterns of dye transfer reported here for the fully mature organ of Corti (at post natal day (P)20) confirm our previous results obtained from animals at the time of hearing onset (P12). These demonstrated extensive intercellular communication not only in the radial direction, that would create the pathways that would be shortest for the removal of K^+^ from the sensory epithelium, but also longitudinally which could allow the supporting cell syncitia to act as ion buffering reservoirs within the organ of Corti. The present results also confirm that in the mature organ of Corti there are two separate compartments of intercellular communication either side of the pillar cells. Following hair cell loss and the concomitant repair of the sensory epithelium as supporting cells expand, there was no change in the patterns of connexin expression nor of intercellular communication pathways. The functional syncitia amongst supporting cells, that would enable signaling between them, and the compartmentalization are maintained. The remodeling of the epithelium that subsequently occurs appears to be initiated, and to involve exclusively, only the outer compartment at first (that is from the Deiters' cells outwards). The inner compartment of coupled cells, that which is associated with the IHC, is initially unaffected. The inner phalangeal and inner border cells expand to close the lesion when an IHC is lost but they do not appear to spread or migrate as do the cells on the lateral side of the pillar cell partition. This difference in response either side of the pillar cells indicates that the compartments defined by pathways of intercellular communication are functionally separate during the repair processes that follow from hair cell loss.

### Transition to squamous-like epithelium

The transition to the squamous-like epithelium involves predominantly an inward migration of cells from the outer edges of the organ of Corti strip. These movements are distinctly polarized. They proceed in a radial direction across the organ of Corti. In addition to the movement of the cells over the top of the Deiters' cells to meet the pillar cells, which in part is achieved through a partial collapse of the Deiters' cells to create a folding of the organ of Corti that brings the Hensen's cells closer to the intact erect pillar cell, there is also movement of cells along the basilar membrane. The presence of Deiters' cells within the tunnel of Corti seems to involve the directed spreading of the bodies of these cells through the spaces between the phalangeal processes of adjacent outer pillar cells. In many tissues the substrate to generate forces for directed cell spreading and movement is provided by asymmetric distribution of filamentous(f)-actin accumulations at cell junctions, polarized to particular sides of the cell, , in association with myosin II [52], [53], [54]. Polarised accumulations of f-actin at the junction between the Hensen's cells and third row Deiters' cells suggest Hensen's cells may be at a leading edge in the initial stages. An actin rich band was also evident along the junction between the outer and inner segments of the flattening epithelium. This has an appearance similar to an actin “purse string” that has been identified, with associated myosin, as the driving component for wound closure in epithelial sheets [52], [55]. Thus, the closing of the “wound” across the organ of Corti may be accomplished in a similar manner. In fact the overall pattern of re-organisation bears distinct similarities to the cell movements associated with wound healing in skin. Cytokines and other similar factors released by inflammatory cells provide signaling molecules that direct and regulate wound healing responses in many tissues [53], [56], [57]. However, we could not detect the involvement of macrophages or other inflammatory cell types in association with the cell migratory phase of “healing” of the damaged cochlear sensory epithelium. Further studies to identify the nature and source of the signaling molecules that mediate these active cell movements are now required.

The re-organisation of the organ of Corti also bears some similarities with squamous metaplasia that occurs, for example, during repair of airway epithelia after injury [58]. Here, where the columnar ciliated and secretory epithelial cells are damaged and expelled from the epithelium, cuboidal basal cells, which are normally intercalated between the columnar cells, migrate along the basement membrane and fill the spaces from which the columnar cells have been lost and form a squamous epithelium covering the damaged area. Proliferation and subsequent re-differentiation of these cells restores the epithelium. There is also evidence that ciliated cells, considered normally to be terminally differentiated, may transdifferentiate directly to squamous cells and contribute to the regenerated epithelium [59]. This raises the possibility that in the remodeled organ of Corti, transdifferentiation of the columnar supporting cells to less specialized squamous-like cells contribute to the population of cells forming the flattened epithelium. However, we could find no direct evidence that this occurred. With the collapse of the phalangeal processes of Deiters' and pillar cells, the retained cell bodies resulted in cuboidal-like cells resting on the basilar membrane but these appeared to retain obvious specializations, most notably the organized microtubular and microfibrillar cytoskeletal components. At the edges of those regions where the flattened epithelium developed, there was no clear evidence of intermediate stages between cells with cytoskeletal specialisations of differentiated Deiters' or pillar cells and the cuboidal like cells which exhibited relatively little cytoplasmic specialization. If this is so, it would suggest that the Deiters' and pillar cells most likely die. Counts of the number of nuclei across the basilar membrane from the spiral limbus to the lateral wall suggest the number of cells comprising the flattened epithelium is less than the number across the basilar membrane in the immediate aftermath of hair cell loss when differentiated Deiters' and pillar cells are still present. This suggests that some cell death may occur during the re-modelling of the epithelium. One previous report has described what appeared to be apoptosis of Deiters' cells [60]. We did not detect any nuclei with apoptotic characteristics during the remodeling phase nor was there evidence of cellular debris attributable to degenerating supporting cells within or around the cells in the developing squamous-like epithelium. However, the death of a cell and the removal of the debris will take only a few hours whereas the re-organisation of the epithelium occurs on much longer time scales and only a very few cells will die. Consequently, capturing the individual cell death events in a “snapshot” of an ongoing process that is occurring in a small area of tissue over a prolonged time scale is difficult. Taken all together therefore, we conclude that Deiters' and pillar cells - i.e. those cells that were initially in contact with the hair cells and are equivalent to cells in the sensory epithelia of the ear in other vertebrate classes from which replacement hair cells are generated - eventually die. We are now seeking further confirmation of that.

The cells that comprise the flattened epithelium show few particular cytoplasmic specializations. Subcellular organelles are quite sparse, there are no prominent cytoskeletal assemblies and the tight-adherens junctions at the cells' apices are simple, with none of the extensive developments that are associated with Deiters' and pillar cells. They do, however, appear to retain, at least initially, unusually large gap junctions that contain both Cx26 and Cx30 often apparently in the same gap-junctional plaque suggesting heteromeric channel conformations [33] Overall the characteristics closely resemble those of the Claudius cells that normally lie to the outer side the organ of Corti strip and cells of inner sulci of the undamaged organ of Corti. It seems reasonable to conclude therefore that it is the spreading and flattening of these cells which forms a major component of the flattened epithelium.

### Implications for regenerative therapies

The results of this analysis of the epithelial remodelling in the organ of Corti do, however, demonstrate several features that may have implications for possible regenerative therapies aimed at replacing lost hair cells. First, there is no evidence of significant de-differentiation or reversion to a less mature state of Deiters' cells, pillar cells, inner phalangeal or inner border cells. These are the supporting cells that would most likely be considered as the source for generating new hair cells intrinsically since the equivalent, though less specialized, cells in inner ear epithelia of non-mammalian vertebrates, and indeed the mammalian vestibular system, are those from which regenerated hair cells arise. Most of the particular functional specializations of the Deiters' and pillar cells that are acquired during late stages of organ of Corti maturation persist for a remarkably long time after hair cell loss and into the phases of re-organisation. It has also been found that Deiters' cells continue to express Sox2, which is expressed by these cells in the mature organ of Corti, for months after hair cell loss and no evidence for its down-regulation was ever detected [61].

It has been thought that the way in which supporting cells in the organ of Corti differentiate and acquire particular specializations during late stages of maturation is associated with their mitotic quiescence, and that induction of cell division in the damaged mature organ of Corti, if at all possible, may require that first these cells de-differentiate to a more immature phenotype. It has been reported that in the repaired mature organ of Corti containing Deiters' and pillar cells, supporting cells can be induced to convert into hair cells following transfection with the Atoh-1 gene [14]. However, there is some controversy over that report [16] and the findings have not yet been replicated elsewhere. Consequently there is a continuing belief that induction of conversion of supporting cells to hair cells using therapies that derive from the expression of single genes or fate determination pathways that are usually active at quite early stages of inner ear development may also require that supporting cells assume a status closer to that at those developmental stages. Thus, the continuing differentiation of supporting cells in the mature organ of Corti following hair cell loss may be an impediment to inducing hair cell regeneration intrinsically. The supporting cells in the repaired epithelium also retain highly developed adherens junctions as shown by the persistence, until replacement by the squamous-like epithelium, of wide bands of intense labeling for actin around the apices of Deiters' cells and the actin labeling in pillar cells. Such junctions are likely to be difficult to dissociate to allow incorporation of cells from extrinsic sources, such as hair cell precursors derived from stem cells, into the sensory epithelium. It is also possible that continuing maturity of the supporting cells may not be a conducive environment for nurturing the differentiation of hair cell precursors into mature hair cells [20]. Thus, the persistence of mature, differentiated supporting cells in the repaired organ of Corti following hair cell loss may be a consideration for the use of stem cell-based therapies at this stage of the epithelial re-organisation.

The second finding of relevance to hair cell regeneration strategies is that transition from a columnar epithelium to the squamous-like epithelium occurs initially in patches apparently randomly distributed along the organ of Corti. This results in segments of squamous-like epithelium interspersed with segments of repaired columnar epithelium comprised of Deiters' and pillar cells. While eventually the squamous-like epithelium may come to predominate, the patch-like condition persists for some time. This condition is not the consequence of the treatment regime used to initiate hair cell loss. We (Taylor and Forge, unpublished observations) have found this discontinuous development of squamous-like epithelium in both a strain of mouse in which the gene encoding Ptprq, which is normally expressed in hair cells, has been knocked out resulting in progressive loss of hair cells in a base-to apex pattern [62], and following hair cell loss in animals in which Cx26 has been conditionally ablated from supporting cells [63] or which express a dominant negative mutation of the gene encoding Cx26 [64]. Similar patch-like development of squamous-like epithelium also occurs in the organ of Corti of guinea pigs following chronic gentamicin treatment (e.g. fig. 1 in [9], [65]). Thus the progression of re-organisation reported in this paper seems to be a general phenomenon irrespective of the initial trigger for hair cell loss or the species affected, and although further detailed studies of human temporal bones are essential, there is some evidence that variability of the pathology along the organ of Corti may exist in the cochleae of some profoundly deaf patients, those who might be considered to benefit from a hair cell regenerative therapy. In terms of applying such a therapy, variability of the cellular environment along an individual organ of Corti may pose problems, since different types of potential regenerative therapy are likely to require different cellular substrates upon which to work. Variability of that substrate in an individual cochlea may require the application of more than one strategy.

The third observation that might be of significance for regenerative therapies is that the speed and extent of transition from the repaired organ of Corti to the squamous epithelium is affected by the genetic background. Whereas in both the C57BL/6 animals and the CBA/Ca strain loss of almost all OHC and the formation of the repaired organ of Corti occurred within the first 48 hours of treatment, the formation of the squamous-like epithelium occurred much sooner in C57BL/6 mice than in CBA/Ca. In the latter strain, the repaired columnar organ of Corti with Deiters' and pillar cells persisted for several months. This variability is not related to the nature of the damaging insult. We, again, (Taylor, Richardson and Forge, unpublished) have observed that in Ptprq^−/−^ animals on a C57BL/6 background, areas of squamous-like epithelium were evident by 6–7 weeks of age, whereas in older animals with a CBA/Ca background despite extensive hair cell loss there was no squamous-like epithelium. In C57BL/6, a “natural” loss of hair cells occurs quite early in life, beginning at around 6–8 months of age, whereas the CBA/Ca mice retain hair cells for at least 24 months [41], [66], [67]. However, the timing of the initiation of formation of the squamous-like epithelium does not seem to be related directly to hair cell loss *per se*. As pointed out above, in the present study the induced loss of OHC occurred to the same extent, and over the same time frame, in both strains. Furthermore, while in both strains loss of IHC was delayed relative to that of OHC loss, initiation of transition to the “flat” epithelium was not dependent upon loss of IHC. In both strains transition was evident when IHC were still present and indeed IHC may themselves have become victims of the re-organisation process. At the same time, complete loss of both OHC and OHC could persist without the initiation of remodelling. Thus, there would appear to be genetic factors that influence how rapidly organ of Corti re-modelling proceeds. If this is also true in the human population then there would be differences between individuals in the cellular nature of the organ of Corti in patients who have suffered extensive hair cell loss and have been deaf for some time. This further emphasizes the need for examination of human temporal bones from profoundly deaf patients. This likely variability between individuals, coupled with the possibility of variability in the cellular nature of the epithelium covering the basilar membrane in an individual cochlea, discussed above, poses complications for assessing the most suitable regenerative strategy for a particular patient. There is as yet no way of determining the likely status of the organ of Corti inside the cochlea from the auditory testing procedures used to assess patients.

To conclude, the results from this study demonstrate that during the initial stages of repair Deiters' cells retain the majority of their specialisations, with the exception of Kir 4.1, an environment that may well provide some support for the survival of any replacement hair cells. However, the presence of differentiated Deiters' and pillar cells may hinder endogenous hair cell regeneration by phenotypic conversion of supporting cells like that which occurs in the mammalian vestibular system. The variability of re-modelling and the migration of Hensen's and Claudius cells towards the pillar cells that results in the development of regions of flat epithelium along the cochlea suggests that more than one strategy may be required to re-create a functioning cochlea, depending upon the nature of the lesion. The rate, and thus the extent, of remodeling may well be influenced also by the genetic background adding a further complication to assessing appropriate regenerative therapies.

## Supporting Information

Figure S1 **Early re-organisation of Deiters' cells.** **A–C SEM** Stereopair images displayed as (red/blue) anaglyphs. Scale bars: 10 µm. **A. Undamaged organ of Corti.** Basal coil of the cochlea, broken open at the level of the third row Deiters' cells. The phalangeal processes (arrows) of the Deiters' cell rise at an angle from the long axis of the cell body (Dc) in the longitudinal direction along the organ of Corti to the luminal surface one-two OHC away. Scale bar: 10 µm. **B. C57BL/6; 24 h post-treatment.** Basal coil. Where OHC persist, the phalangeal process of those Deiters' cell that surround the OHC base are at an angle to the long axis of the cell body; where the OHC are lost, the phalangeal process rises straight up from the cell body (arrow). OHC in 3^rd^ (ohc3) and 2^nd^ (ohc2) row exposed. **C. C57BL/6. Apical coil.** The phalangeal processes of the Dieters' cells are in line with the long axis of the cell body. **D. CBA/Ca. 7 days post-treatment.** TEM of thin section of 1^st^ row Deiters' cells showing nucleus migrated towards the apical end of the cell and the cell body rounded. The arrow indicates prominent microtubules, which together with the dense, deep and wide microfilament assembly at the apical intercellular junction, identify the cell as a Deiters' cell. Scale bar: 5 µm.
(TIF)

Figure S2 **Thin sections showing appositions of Deiters' cell where phalangeal processes have expanded.** No gap junctions are evident along these regions of close apposition. **A.** Expanded phalangeal processes of two Deiters' cell that have become closely adjacent to obliterate the normal extracellular space (of Nuel). **A'** The plasma membranes of the two cells in the region of close apposition at higher power. For orientation, the arrow in each panel indicates the same structure (an elongated mitochondrion). The membranes of the two cells are closely parallel but there is no evidence of a meeting between the membranes characteristic of gap junction plaques. Scale bars: A, 2 µm; A', 0.5 µm. **B.** Another example of an expanded Deiters cell phalangeal process that has become closely adjacent to the expanded phalangeal processes of Deiters' cells either side. Arrows indicate the extent of the close apposition of the membranes of the adjacent cells along which there no evidence of characteristic morphology of gap junctions in thin sections. The characteristic thin section appearance of a gap junction is seen in the electron density of the membranes that form the vesicular surround of the annular (internalised) gap junction, indicated by the asterisk. (Annular gap junctions are commonly seen in Deiters' cells in undamaged tissue [33]). Scale bar: 1 µm.
(TIF)
