# Supplementary figures and images for: Defining the Cellular Environment in the Organ of Corti following Extensive Hair Cell Loss: A Basis for Future Sensory Cell Replacement in the Cochlea
Source: PLoS One. 2012 Jan 27;7(1):e30577. doi: 10.1371/journal.pone.0030577 (PMC3267727; doi:10.1371/journal.pone.0030577)

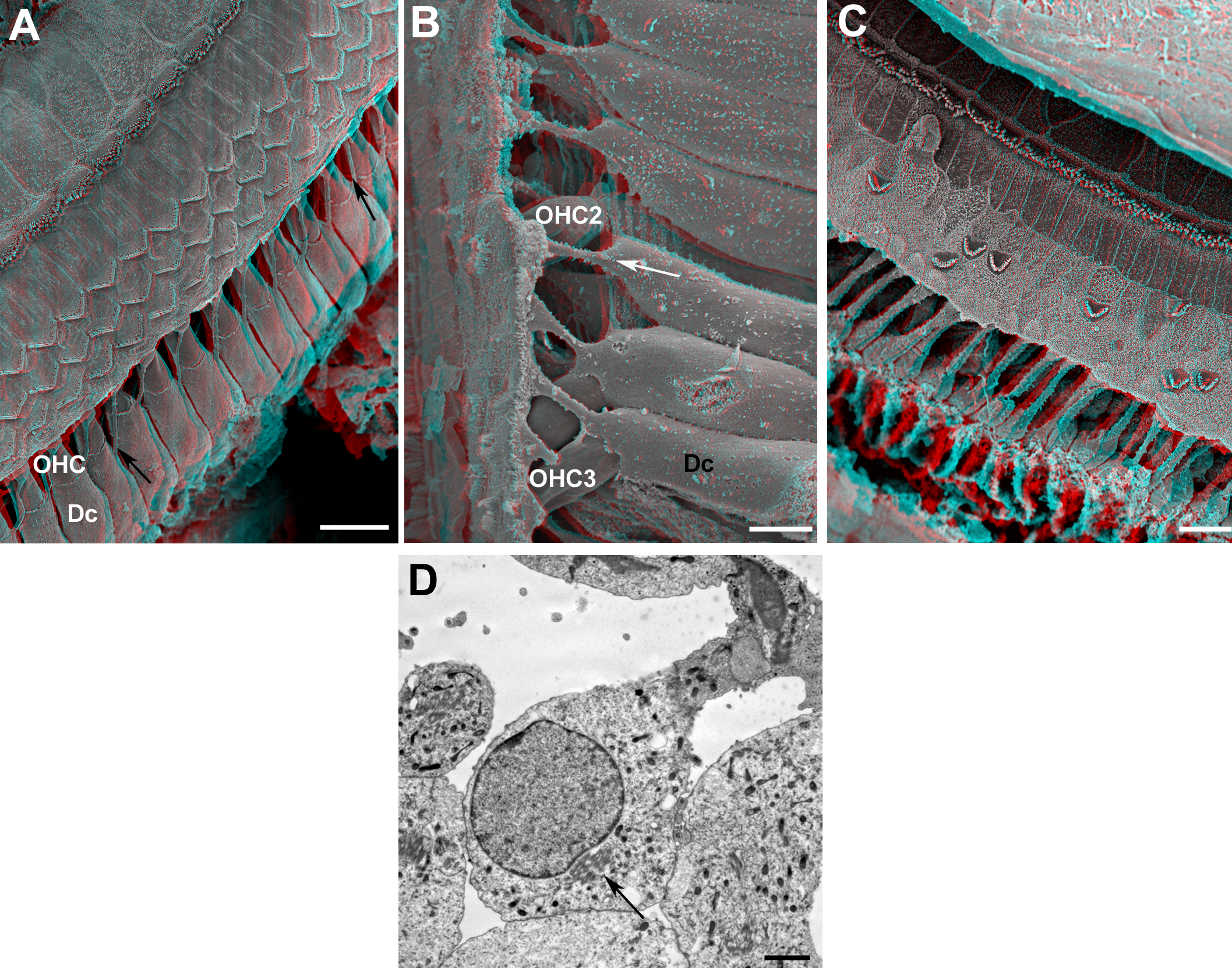

Supplement: Figure S1 — Early re-organisation of Deiters' cells. A–C SEM Stereopair images displayed as (red/blue) anaglyphs. Scale bars: 10 µm. A. Undamaged organ of Corti. Basal coil of the cochlea, broken open at the level of the third row Deiters' cells. The phalangeal processes (arrows) of the Deiters' cell rise at an angle from the long axis of the cell body (Dc) in the longitudinal direction along the organ of Corti to the luminal surface one-two OHC away. Scale bar: 10 µm. B. C57BL/6; 24 h post-treatment. Basal coil. Where OHC persist, the phalangeal process of those Deiters' cell that surround the OHC base are at an angle to the long axis of the cell body; where the OHC are lost, the phalangeal process rises straight up from the cell body (arrow). OHC in 3rd (ohc3) and 2nd (ohc2) row exposed. C. C57BL/6. Apical coil. The phalangeal processes of the Dieters' cells are in line with the long axis of the cell body. D. CBA/Ca. 7 days post-treatment. TEM of thin section of 1st row Deiters' cells showing nucleus migrated towards the apical end of the cell and the cell body rounded. The arrow indicates prominent microtubules, which together with the dense, deep and wide microfilament assembly at the apical intercellular junction, identify the cell as a Deiters' cell. Scale bar: 5 µm. (TIF) [file pone.0030577.s001.tif]

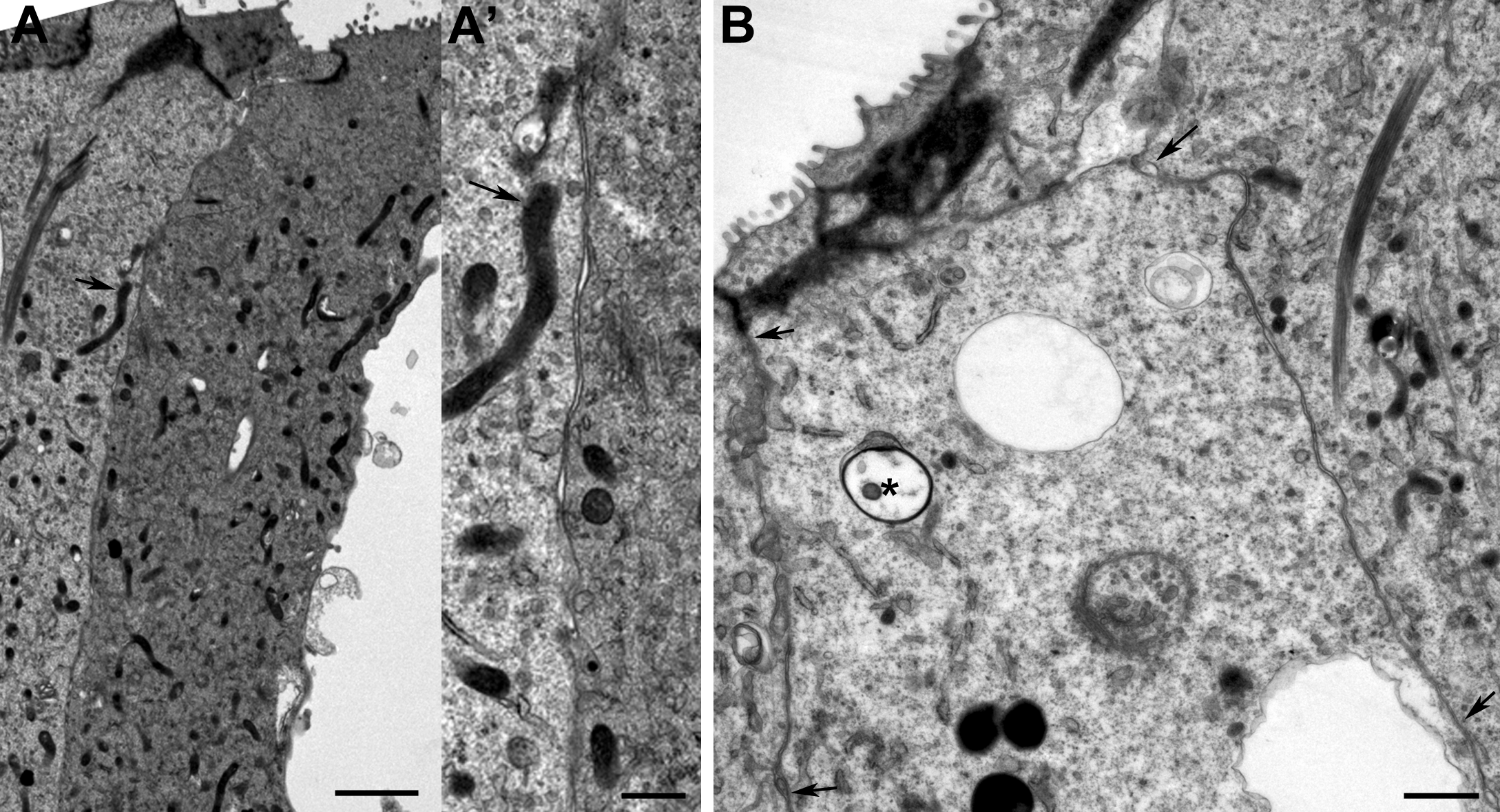

Supplement: Figure S2 — Thin sections showing appositions of Deiters' cell where phalangeal processes have expanded. No gap junctions are evident along these regions of close apposition. A. Expanded phalangeal processes of two Deiters' cell that have become closely adjacent to obliterate the normal extracellular space (of Nuel). A' The plasma membranes of the two cells in the region of close apposition at higher power. For orientation, the arrow in each panel indicates the same structure (an elongated mitochondrion). The membranes of the two cells are closely parallel but there is no evidence of a meeting between the membranes characteristic of gap junction plaques. Scale bars: A, 2 µm; A', 0.5 µm. B. Another example of an expanded Deiters cell phalangeal process that has become closely adjacent to the expanded phalangeal processes of Deiters' cells either side. Arrows indicate the extent of the close apposition of the membranes of the adjacent cells along which there no evidence of characteristic morphology of gap junctions in thin sections. The characteristic thin section appearance of a gap junction is seen in the electron density of the membranes that form the vesicular surround of the annular (internalised) gap junction, indicated by the asterisk. (Annular gap junctions are commonly seen in Deiters' cells in undamaged tissue [33]). Scale bar: 1 µm. (TIF) [file pone.0030577.s002.tif]
